# Surrogate-Assisted Inverse Design of the Power-Law Index in Axially Functionally Graded Fluid-Conveying Pipes for Target Modal and Deflection Performance

**DOI:** 10.3390/ma19183936

**Published:** 2026-09-16

**Authors:** Lun Gao, Jijun Gu, Tianjin Guo, Shanshan Zhao, Junjie Li

**Affiliations:** 1 School of Safety Science and Engineering, Xinjiang Institute of Engineering, Urumqi 830023, China; gl07@xjie.edu.cn (L.G.); gtj@xjie.edu.cn (T.G.); shanshanzhao@xjie.edu.cn (S.Z.); ljj@xjie.edu.cn (J.L.); 2 Xinjiang Key Laboratory of Coal Mine Disaster Intelligent Prevention and Emergency Response, Xinjiang Institute of Engineering, Urumqi 830023, China; 3 College of Mechanical and Transportation Engineering, China University of Petroleum–Beijing, Beijing 102249, China

**Keywords:** inverse design, multi-layer perceptron, functionally graded pipe, pipe conveying fluid, particle swarm optimization, generalized integral transform technique

## Abstract

A surrogate-assisted inverse design framework is presented for selecting the power-law index of clamped–clamped axially functionally graded (AFG) Timoshenko pipes conveying fluid. The GITT model, 336-sample database, and trained MLP forward surrogate were developed in our previous study; the present contribution begins with the formulation and solution of the inverse problem. Millisecond-speed MLP inference is embedded in grid, particle swarm optimization (PSO), and genetic algorithm (GA) searches for prescribed modal-frequency and deflection targets. Single-variable, two-variable, weighted-sum, constrained, and Pareto formulations are examined. Continuous candidates from the single-variable cases are subjected to GITT-database interpolation verification, which is explicitly distinguished from a new independent GITT calculation. The feasible single- and dual-modal examples produce small database-interpolated target residuals, whereas an intentionally unattainable triplet case retains an approximately 16% fundamental-frequency residual and demonstrates the need for feasibility screening. The two-variable maps reveal non-unique parameter couplings and are treated as exploratory surrogate results unless the complete candidate coincides with, or is independently assessed against, the available database. A five-network ensemble assesses repeatability with respect to training-data partitioning only, while a sensitivity analysis connects the selected indices to the high-sensitivity gradation range. In the synchronized Case A benchmark, the online MLP grid and PSO searches require approximately 0.044 and 0.210 s, respectively, excluding the inherited offline GITT-database construction cost.

## 1. Introduction

Axially functionally graded (AFG) pipes conveying fluid offer tunable stiffness and mass distributions through continuous axial gradation, with the power-law index *k* serving as a primary design parameter governing natural frequencies, critical flow velocities, and vibration amplitudes [1,2,3,4,5,6,7]. In engineering practice, designers must often answer an inverse question: given fixed geometry and flow conditions together with target modal frequencies and/or a deflection limit, which gradation index *k* should be selected? Timoshenko-based models and recent studies on AFG fluid-conveying pipes have clarified the forward sensitivity of dynamic responses to *k*, *u*, and L/D [8,9,10,11,12,13], but systematic surrogate-assisted inverse formulations for this pipe class remain limited.

For an as-built pipe, dimensional tolerances, ovality, wall-thickness variation, and surface roughness can alter both structural stiffness and hydraulic resistance. Roughness and deviations in the bore geometry increase frictional pressure loss and may modify the relationship among pressure, mean velocity, and fluid temperature; the magnitude also depends on fluid density and viscosity and on whether the internal flow is laminar, transitional, turbulent, single-phase, or multiphase [8,9]. These hydraulic and thermo-fluid effects can therefore shift the operating point supplied to a vibration model. The present inverse framework deliberately isolates the structural-dynamic problem: it assumes single-phase incompressible water of constant density and a prescribed spatially uniform mean velocity, and it does not solve the pressure-drop, temperature, viscosity, or roughness fields. Consequently, those quantities do not enter the GITT or MLP convergence criteria directly; their influence is represented only if they change the prescribed velocity or the structural/material parameters.

Previously, the authors developed and validated a physics-based GITT benchmark coupled with an MLP surrogate for the forward vibration analysis of Alumina–Steel AFG Timoshenko pipes conveying fluid [14]. That study established a parametric database of $N_{s}=336$ high-fidelity GITT samples over (k,u,L/D)∈[0,10]×[0,6.2]×[5,100], trained three decoupled feedforward MLP networks achieving $R^{2}>0.99$ on an independent test set, and demonstrated a computational speedup of $\eta \approx 2.9\times 10^{5}$ for $10^{4}$ parametric evaluations relative to sequential GITT. The present work builds directly on that forward pipeline to address the inverse problem: given target dynamic performance, what is the optimal *k*?

High-fidelity GITT solvers provide reliable benchmarks for coupled fluid–structure dynamics [4,15,16,17], yet each parametric evaluation is computationally expensive when *k* must be searched repeatedly  [18,19,20]. Surrogate-assisted metamodels therefore offer a practical route to acceleration [21,22,23]. Existing inverse studies on functionally graded structures often rely on reduced models, manual sweeps, or direct high-fidelity search with limited budgets  [18,24,25,26]. Despite these advances, three gaps remain. **First,** existing AFG pipe studies focus almost exclusively on forward parametric analysis—quantifying how *k*, *u*, and L/D influence vibration responses—whereas the automated inverse problem of determining the gradation index that achieves prescribed vibration targets has not been addressed. **Second,** surrogate-assisted inverse studies do not always distinguish the optimizer residual from the discrepancy obtained through a database-based reference assessment; explicitly reporting the assessment source and its limitations is therefore essential. **Third,** no existing work simultaneously performs single-variable and two-variable multi-objective Pareto optimization for AFG pipes, and the coupled trade-offs among material gradation, flow conditions, and geometric parameters remain unexplored. The present study addresses these inverse-design questions by reusing the published GITT–MLP forward model and adding target-driven optimization, feasibility, and trade-off analyses.

The main contributions are as follows:

- **Inverse-design layer built on an inherited forward model.** The previously published GITT solver, database, and trained MLP surrogate [14] are reused without being claimed as new; the present work formulates the target-to-design problem and couples the frozen surrogate to grid, PSO, GA, constrained, and Pareto searches. Continuous single-variable candidates are subsequently subjected to GITT-database interpolation verification.
- **Multi-case inverse design.** Weighted-sum, constrained, and Pareto formulations are applied to single-modal (Case A), dual-modal (Case B), deflection-aware (Case C), and Pareto trade-off (Case D) targets.
- **Two-variable extension.** The framework is extended to simultaneous optimization of (k,u) and (k,L/D), demonstrating scalability beyond the single-variable baseline and revealing the attainable performance envelopes.
- **Constraint consistency, training-repeatability, and sensitivity interpretation.** An inactive-constraint case checks that the constrained implementation reduces to the unconstrained solution when the bound is not active; a five-network ensemble assesses variability due only to training and data partitioning; and a sensitivity analysis relates the reported $k^{★}$ values to the high-sensitivity gradation range.

The remainder of this paper is organized as follows. Section 2 summarizes the forward model and MLP surrogate from [14]. Section 3 formulates the inverse problem and describes the optimization methodology. Section 4 presents single-variable and two-variable case studies. Section 5 provides GITT-database interpolation verification and computational performance analysis. Section 6 discusses implications, sensitivity–inverse design connections, and limitations, and Section 7 draws conclusions.

## 2. Forward Model and MLP Surrogate (Summary)

The forward pipeline—physical model, GITT solution, and MLP training—is fully developed and validated in [14]. Only the elements essential for the inverse design are recapitulated here. The GITT formulation, the 336-sample database, the train–test partition, and the three trained MLP predictors are inherited components rather than methodological contributions of the present manuscript. The new work begins with the inverse formulations in Section 3.

**Figure 1:**
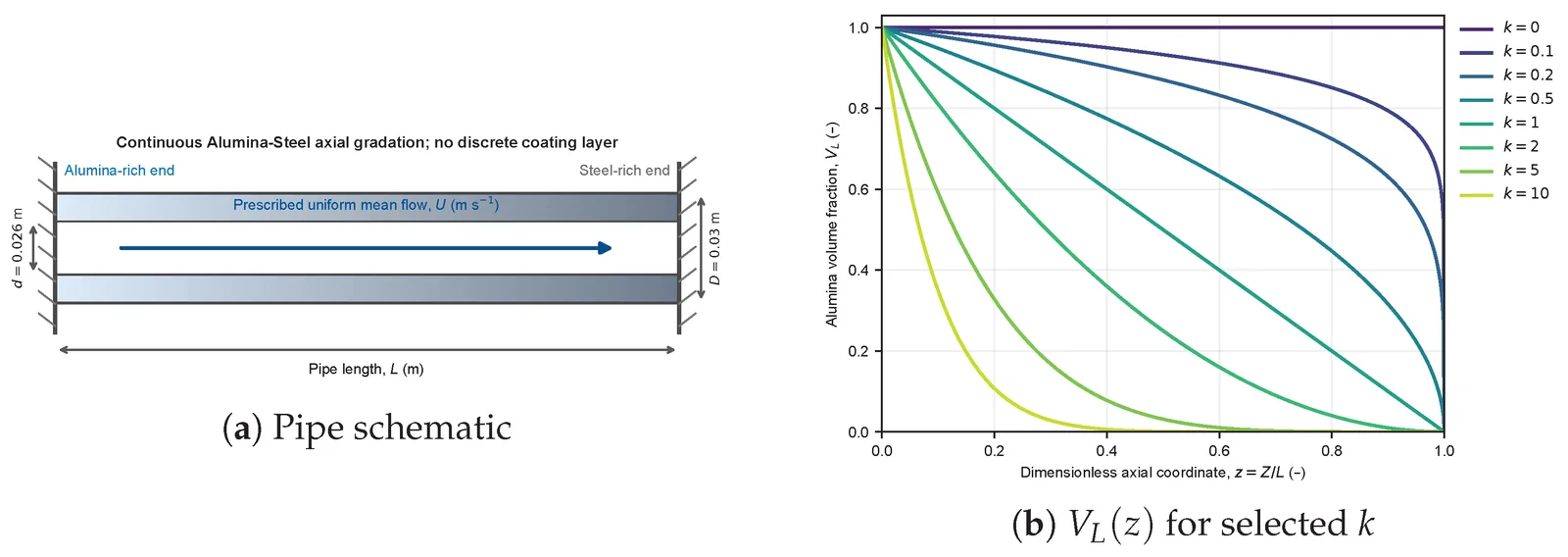
Clamped–clamped AFG Timoshenko pipe with nominal dimensions, prescribed uniform mean flow, continuous Alumina–Steel gradation, and no discrete coating (**a**); dimensionless alumina volume-fraction profiles (**b**). Both panels were redrawn as vector graphics from the model reported in [14].

Figure 1 depicts the clamped–clamped Alumina–Steel AFG Timoshenko pipe conveying fluid. Material properties follow the power-law rule $P\left(Z\right)=P_{L}+\left(P_{R}-P_{L}\right){\left(Z/L\right)}^{k}$ with alumina at z=0 ($E_{L}=390$ GPa, $\rho_{L}=3960$ kg/m^3^) and steel at z=1 ($E_{R}=210$ GPa, $\rho_{R}=7800$ kg/m^3^) [4,14]. Here, *Z* (m) is the axial coordinate, *P* denotes the graded elastic modulus or mass density, and $P_{L}$ and $P_{R}$ are its end-member values. The nominal outer and inner diameters are D=0.030 m and d=0.026 m, respectively. The wall is a continuous Alumina–Steel axial gradation rather than a substrate with a discrete coating; no separate coating thickness or coating property is included. The internal fluid is constant-density water ($\rho_{f}=1000$ kg/m^3^) moving at the prescribed uniform mean velocity *U* (m/s). The formulation contains no independent viscous, structural, or foundation-damping coefficient, so the reported natural frequencies are those of the corresponding undamped linearized system. The GITT solves the coupled Timoshenko equations with N=20 modal truncation using an adaptive stiff ODE integrator (DIVPAG, tolerance $10^{-6}$); parametric sweeps over k∈{0,0.1,…,10}, u∈[0,6.2], L/D∈[5,99] produce $N_{s}=336$ samples [14,17]. A single GITT query at L/D=50 costs $T_{\mathrm{GITT}}\approx 122$ s.

Here, z=Z/L denotes the dimensionless axial coordinate, so that the material endpoints remain z=0 and z=1. The dimensionless variables inherited from the forward model [14] are

(1) $$t=\frac{T}{L^{2}}\sqrt{\frac{E_{0}I_{p}}{\rho_{0}A_{p}+m_{f}}},\;u=UL\sqrt{\frac{m_{f}}{E_{0}I_{p}}},\;y=\frac{Y}{L}.$$

Here, *T* (s) and *Y* (m) are dimensional time and transverse displacement; $E_{0}$ (Pa) and $\rho_{0}$ (kg/m^3^) are the reference elastic modulus and density; $A_{p}$ (m^2^) and $I_{p}$ (m^4^) are the pipe-wall area and second moment of area; $m_{f}=\rho_{f}\pi d^{2}/4$ (kg/m) is the internal-fluid mass per unit length. Accordingly, *k*, *u*, L/D, $\omega_{i}$, and $y_{M}$ are dimensionless. A dimensionless angular frequency is converted through

(2) $$\Omega_{i}=\frac{\omega_{i}}{L^{2}}\sqrt{\frac{E_{0}I_{p}}{\rho_{0}A_{p}+m_{f}}}\;\left(\mathrm{rad}/s\right),\;f_{i}=\frac{\Omega_{i}}{2\pi }\;\left(\mathrm{Hz}\right).$$

Three independent MLP networks (hidden [45,25], tanh, L-BFGS) are trained on a 270/66 interior block-wise split to map $x={\left[k,u,L/D\right]}^{T}\mapsto y={\left[\omega_{1},\omega_{2},y_{M}\right]}^{T}$ with min–max input normalization and log-transformed outputs [14]. Table 1 reproduces the key accuracy metrics: $R^{2}>0.99$ on the independent 66-sample test set, maximum relative errors below 9%, and the published inference time $T_{\mathrm{MLP}}=0.085$ ms/query. For any x within k∈[0,10], u∈[0,6.2], L/D∈[5,100], the surrogate provides $\hat{y}=f_{\mathrm{MLP}}\left(x\right)$ in sub-millisecond time, enabling the rapid inner-loop queries that drive the inverse optimization in Section 3 and Section 4.

The principal symbols, definitions, and units used in the forward and inverse formulations are summarized in Table 2.

Table 2 summarizes the principal notations and units used in the forward and inverse formulations; symbols specific to individual optimization algorithms are additionally defined at their first occurrence below.

### Comparison with a Published Limiting-Case Benchmark

A targeted literature search did not identify a directly matching study that determines the power-law index from prescribed vibration targets for a clamped–clamped AFG Timoshenko pipe conveying fluid. A one-to-one comparison of the inverse solution $k^{★}$ is therefore not available. Instead, the inherited forward model was assessed under a common homogeneous, zero-flow limiting condition using the clamped–clamped Euler–Bernoulli/GITT results of An and Su [27], whose nondimensional frequency definition is identical to that adopted here. At k=0, u=0, and L/D=50, the present Timoshenko–GITT database yields $\omega_{1}=22.2006$ and $\omega_{2}=60.950$, compared with 22.373 and 61.673, respectively, in the published benchmark (Table 3). The corresponding relative differences are 0.77% and 1.17%. The small downward shifts are consistent with the shear-deformation and rotary-inertia effects retained by Timoshenko theory at finite slenderness but omitted by the Euler–Bernoulli model. The present model also reproduces the published qualitative trend that modal frequencies decrease as flow velocity increases and vary systematically with the material-gradation exponent. Thus, this comparison assesses the common forward limiting behavior; it is not presented as a literature validation of the new target-to-*k* inverse formulation.

## 3. Inverse Design Methodology

### 3.1. Inverse Problem Formulation

For a fixed operating condition $\left(u_{0},{\left(L/D\right)}_{0}\right)$ and a prescribed target response $y^{★}={\left[\omega_{1}^{★},\omega_{2}^{★},y_{M}^{★}\right]}^{T}$, the single-variable inverse design problem seeks

(3) $$k^{★}=\arg\min_{k\in [0,10]}\;J\;\left(k;\;u_{0},\;{\left(L/D\right)}_{0},\;y^{★}\right),$$

where J is a scalar or vector objective functional. The two-variable extension searches over (k,u) or (k,L/D):

(4) $$\begin{array}{cc} \left(k^{★},\;u^{★}\right) & =\arg\min_{k\in [0,10],\;u\in [0,6.2]}\;J\;\left(k,u;\;{\left(L/D\right)}_{0},\;y^{★}\right), \end{array}$$

(5) $$\begin{array}{cc} \left(k^{★},\;{\left(L/D\right)}^{★}\right) & =\arg\min_{k\in [0,10],\;L/D\in [5,100]}\;J\;\left(k,L/D;\;u_{0},\;y^{★}\right). \end{array}$$

### 3.2. Objective Functions

The primary scalar objective is a weighted sum of normalized absolute deviations:

(6) $$J_{w}\left(x\right)=\sum_{i=1}^{3}w_{i}\;\Delta_{i}\left(x\right),\;\Delta_{i}\left(x\right)=\frac{|{\hat{y}}_{i}\left(x\right)-y_{i}^{★}|}{|y_{i}^{★}|+\delta },$$

where $w_{i}\geq 0$ with $\sum_{i}w_{i}=1$, and $\delta =10^{-8}$ prevents division by zero. The subscript i∈{1,2,3} identifies $\omega_{1}$, $\omega_{2}$, and $y_{M}$, respectively; a hat denotes an MLP prediction, a superscript star denotes a target, and $w_{i}$, $\Delta_{i}$, δ, and $J_{w}$ are dimensionless. Setting $\left(w_{1},w_{2},w_{3}\right)=\left(1,0,0\right)$ reduces to single-target $\omega_{1}$ inversion.

When constraints are needed, the constrained formulation

(7) $$\min_{x}\;|\omega_{1}^{★}-{\hat{\omega }}_{1}\left(x\right)|\;s.t.\;|{\hat{y}}_{i}\left(x\right)-y_{i}^{★}|\leq \tau_{i},\;\;i=2,3$$

can be used. Here, $\tau_{i}$ is the dimensionless admissible deviation for response *i*. For conflicting targets, the vector objective $J\left(x\right)={\left[\Delta_{1}\left(x\right),\Delta_{2}\left(x\right),\Delta_{3}\left(x\right)\right]}^{T}$ is minimized in the Pareto sense [24].

### 3.3. Optimization Algorithms

Three complementary solvers are implemented:

One-dimensional grid search.

For single-variable optimization of *k*, a uniform grid of $N_{k}=501$ points on [0,10] evaluates $f_{\mathrm{MLP}}$ at negligible cost and serves as the baseline reference solution.

Particle swarm optimization (PSO).

A swarm of $N_{\mathrm{part}}=30$ particles evolves for 80 iterations with Clerc’s constriction factor χ=0.729, cognition and social coefficients $c_{1}=c_{2}=1.49445$ [25]. For two-variable problems, the search space is extended to $R^{2}$ while retaining the same population size and iteration budget.

Genetic algorithm (GA).

A population of 40 individuals evolves for 60 generations with elitism (top 2 retained), blend crossover, and Gaussian mutation ($p_{\mathrm{mut}}=0.15$) [24,26]. For two-variable problems, each individual carries a two-dimensional chromosome (k,v) where *v* denotes the second variable.

Algorithm choice.

Population-based metaheuristics are adopted because the surrogate objective $J_{w}$ can be mildly non-convex when multiple targets are weighted. A fixed iteration budget is used throughout; the convergence histories reported in the numerical case studies show that $J_{w}$ decays sharply within ∼20 iterations. Cross-optimizer agreement on $k^{★}$ supports algorithmic convergence.

Table 4 summarizes the optimizer settings, whereas Algorithm 1 presents the complete inverse-design procedure.
**Algorithm 1** Surrogate-assisted inverse design of *k*, (k,u), or (k,L/D) with database-based candidate assessment.**Input:** Fixed operating parameters, targets $y^{★}$, weights $\left\{w_{i}\right\}$, bounds, trained MLP $f_{\mathrm{MLP}}$.**Output:** Candidate optimum $x^{★}$, GITT-database-interpolated reference response $y_{\mathrm{GITT},\mathrm{db}-\mathrm{int}}\left(x^{★}\right)$ when the required database slice is available.1. Initialize optimizer population/particles within parameter bounds.2. **For** each iteration:    2.1 Evaluate $\hat{y}=f_{\mathrm{MLP}}\left(x\right)$ and compute J via Equation (6).    2.2 Update particles (PSO) or evolve population (GA).    2.3 Continue until the fixed iteration budget is exhausted.3. Select best $x^{★}$ (or Pareto front).4. **Database-based verification:** interpolate the existing GITT database at $x^{★}$ when the complete fixed-parameter slice is available, record $y_{\mathrm{GITT},\mathrm{db}-\mathrm{int}}\left(x^{★}\right)$, identify the assessment method explicitly, and compute target-relative residuals via Equation (8). This step is not a new GITT simulation.


### 3.4. GITT-Database Interpolation Verification Protocol

Because the MLP surrogate is trained on discrete *k* values (k∈{0,0.1,0.2,0.5,1,2,5,10}) while the optimizer treats *k* as continuous, the optimized $k^{★}$ often falls between training points (e.g., $k^{★}=0.23$ or 0.39). For Cases A–D and G, in which *u* and L/D are fixed at sampled database values, the reference response is obtained by piecewise-linear interpolation along the *k* direction within the existing 336-sample GITT database. We call this procedure “GITT-database interpolation verification” to distinguish it explicitly from a newly executed GITT calculation. It provides a database-consistency check at the continuous candidate but is not statistically or numerically independent of the data used to construct the surrogate. For Cases E and F, the optimized second variable does not coincide with a database node; the currently reported database values are therefore identified as nearest-node approximations rather than full interpolation verification. The target-relative residual is

(8) $$\varepsilon_{i}^{\mathrm{db}-\mathrm{int}}=\frac{|y_{i}^{\mathrm{GITT},\mathrm{db}-\mathrm{int}}\left(x^{★}\right)-y_{i}^{★}|}{|y_{i}^{★}|+\delta }\times 100\%,$$

where $y_{i}^{\mathrm{GITT},\mathrm{db}-\mathrm{int}}\left(x^{★}\right)$ denotes a response interpolated from the original GITT database, not a newly computed GITT response. The superscript “db-int” is omitted only in figure axis labels where space is limited.

To complement the global 66-sample test metrics, a local leave-one-out (LOO) analysis was performed around the reported inverse-design candidates. Twelve unique neighboring GITT nodes were selected: the two *k* nodes bracketing each single-variable candidate and the four nearest nodes in normalized active-coordinate space for each two-variable candidate. Each selected node was removed in turn, the three MLPs were retrained on the remaining 335 samples using the same architecture and preprocessing, and the omitted response was predicted. For the common Case A/B/D/G neighborhood, the mean (maximum) relative LOO errors for $\left(\omega_{1},\omega_{2},y_{M}\right)$ were (0.81,0.27,0.59)% [(0.92,0.44,1.10)%]. The corresponding values were (1.83,0.55,0.86)% [(3.28,0.79,1.03)%] near Case C, (0.87,0.65,0.38)% [(1.69,1.17,0.83)%] near Case E, and (1.63,1.33,1.27)% [(2.20,2.39,2.59)%] near Case F. These local errors are generally below the global worst-case errors, but they also show that a near-zero optimizer residual cannot be interpreted as sub-1% forward accuracy in every neighborhood.

## 4. Numerical Case Studies

All cases use the MLP surrogate from Section 2 without retraining. Table 5 and Table 6 summarize the single-variable setup and results; the following subsections present Cases A–G and cite each associated figure in numerical order.

### 4.1. Case A: Target Fundamental Frequency

**Figure 2:**
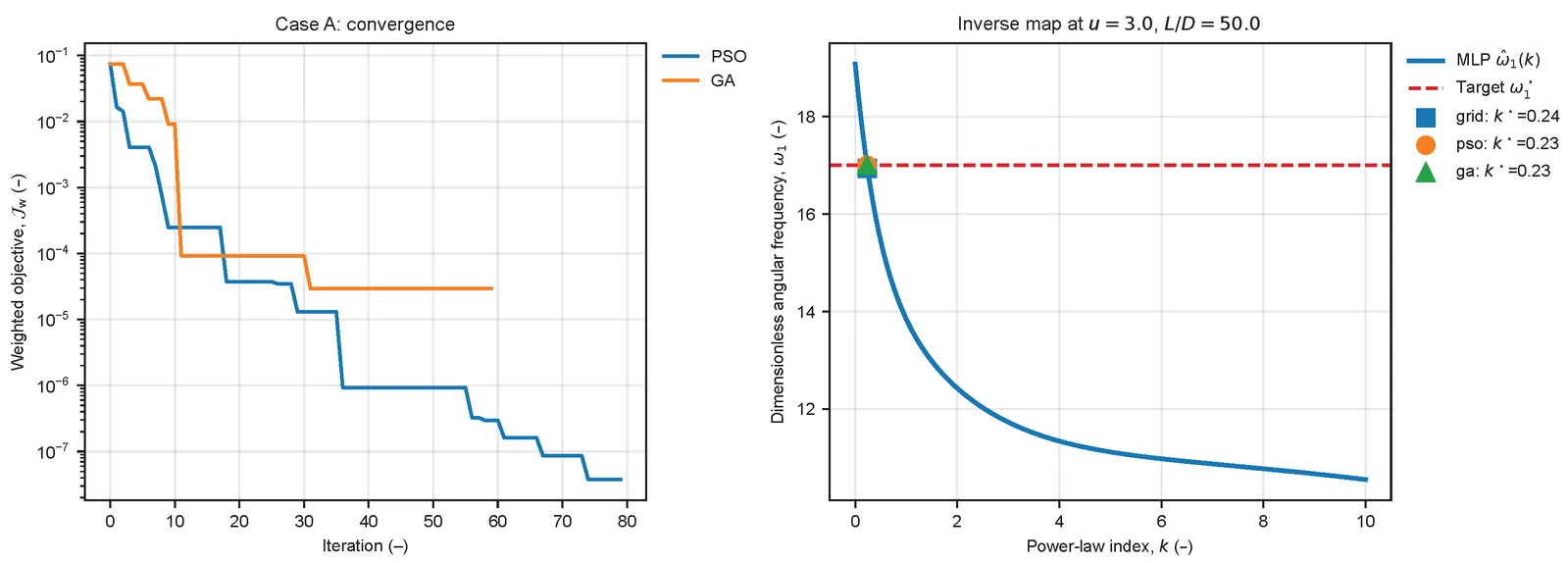
Case A: optimizer convergence (**left**) and MLP forward map ${\hat{\omega }}_{1}\left(k\right)$ with target and optimized $k^{★}$ (**right**).

**Figure 3:**
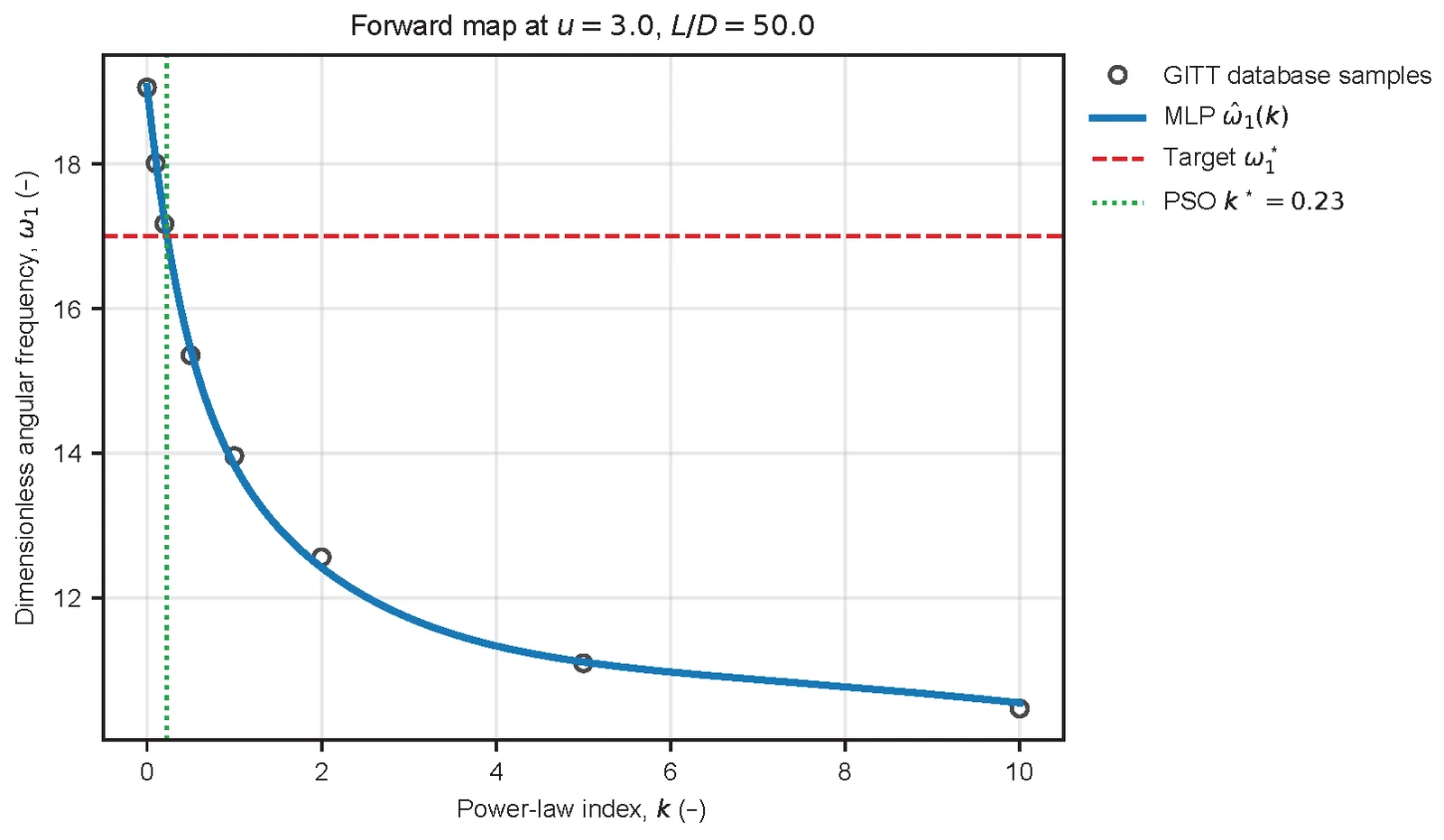
GITT benchmark samples and MLP forward curve at the Case A operating point.

Case A specifies $\omega_{1}^{★}=17.0$ at $u_{0}=3.0$, ${\left(L/D\right)}_{0}=50$, with $w_{1}=1$. Figure 2 shows optimizer convergence and the MLP forward curve ${\hat{\omega }}_{1}\left(k\right)$ with the target and all three optimized designs. Table 6 lists the MLP-optimized designs: all three solvers converge to $k^{★}\approx 0.23$–0.24, yielding ${\hat{\omega }}_{1}\approx 17.0$. GITT-database interpolation verification at the continuous PSO design $k^{★}=0.2303$, using the bracketing nodes k=0.2 and 0.5 rather than a nearest-node tolerance, provides $\omega_{1}^{\mathrm{GITT},\mathrm{db}-\mathrm{int}}=16.991$ and a target-relative residual of 0.055%. Figure 3 overlays the GITT benchmark samples (open circles) with the MLP forward curve (solid line) at this operating point. The MLP faithfully reproduces the decreasing trend of $\omega_{1}$ with increasing *k*, capturing both the steep gradient in the alumina-rich regime (k<1) and the gradual saturation toward the steel-rich limit (k>2). The excellent pointwise agreement across all eight GITT training values of *k* confirms that the surrogate reliably interpolates between training points, which is essential for the inner-loop optimization in Cases A–G.

**Figure 4:**
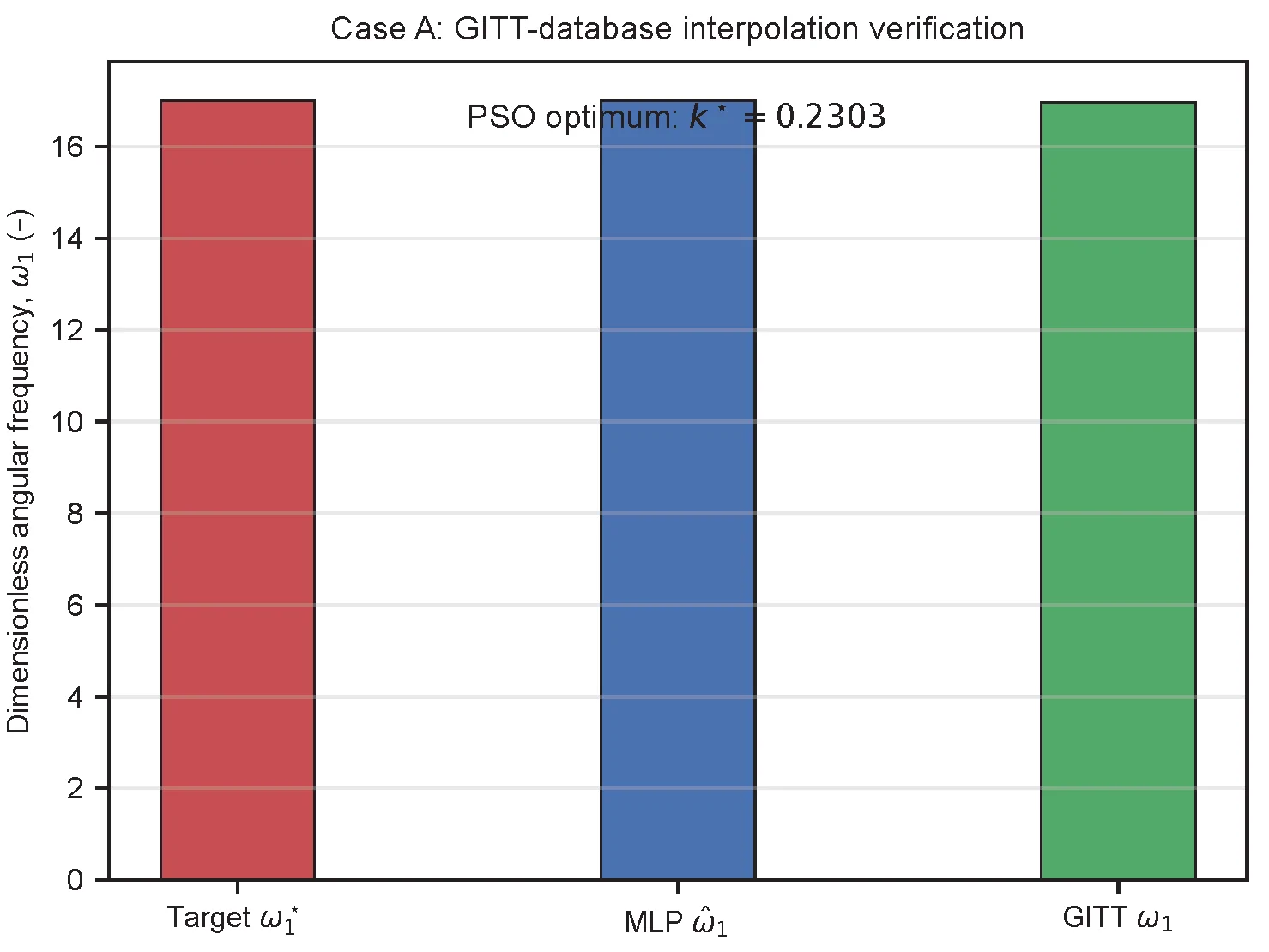
Case A: target, MLP prediction, and GITT-database interpolation verification at the optimized $k^{★}=0.2303$ (PSO design).

The local MLP–database-interpolation gap at $k^{★}=0.2303$ is approximately 0.055%. As $k^{★}$ lies between the training points k=0.2 and k=0.5, the MLP must interpolate. The global maximum test error of 8.90% is not a uniform local uncertainty bound, and the near-zero surrogate objective alone does not imply a comparably small physical target residual. Figure 4 therefore compares the target and MLP prediction with the database-interpolated GITT reference at the PSO candidate. The resulting 0.055% residual is evidence only for consistency with this local database interpolation; it should not be interpreted as an independent high-fidelity certification.

### 4.2. Case B: Simultaneous Modal Targets

**Figure 5:**
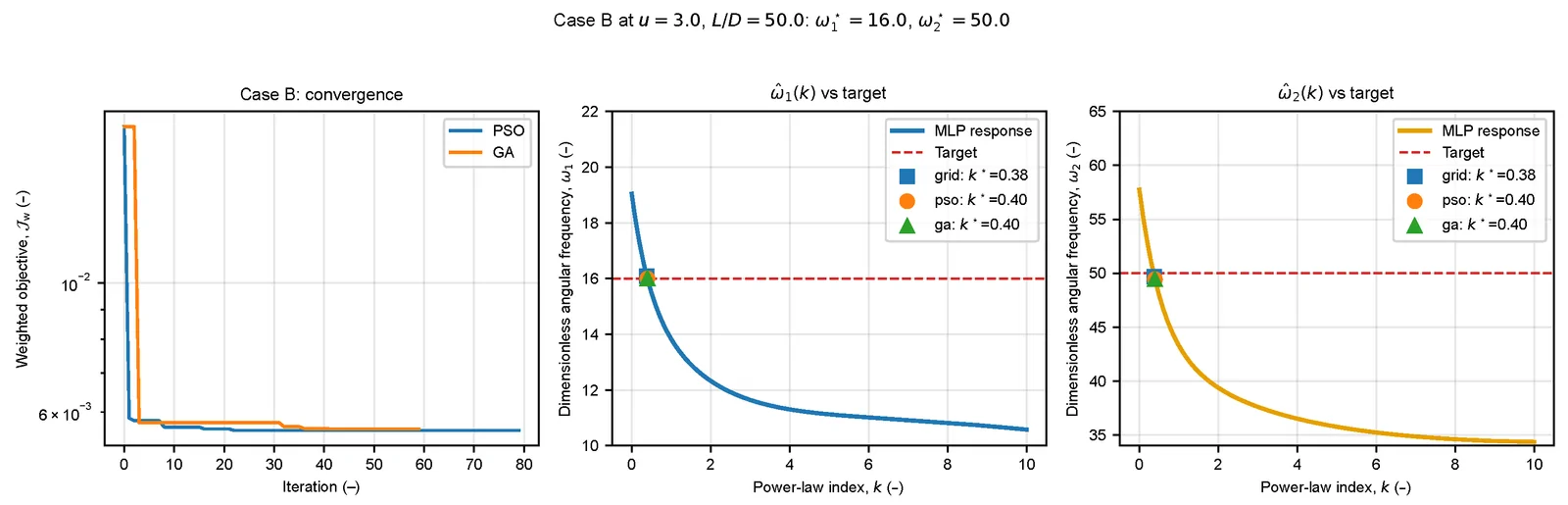
Case B: optimizer convergence (**left**) and dual MLP forward maps ${\hat{\omega }}_{1}\left(k\right)$, ${\hat{\omega }}_{2}\left(k\right)$ with targets and $k^{★}$ (**center**,**right**).

Both $\omega_{1}^{★}=16.0$ and $\omega_{2}^{★}=50.0$ are enforced at $(u_{0},{\left(L/D\right)}_{0})=\left(3.0,50\right)$ with equal weights. Compared with Case A, the lower fundamental target and the explicit second-mode requirement shift the optimal gradation index to $k^{★}\approx 0.38$–0.39, reflecting the stronger tunability of *k* on $\omega_{2}$ identified in [6,10,14]. Figure 5 shows convergence and dual MLP forward maps.

**Figure 6:**
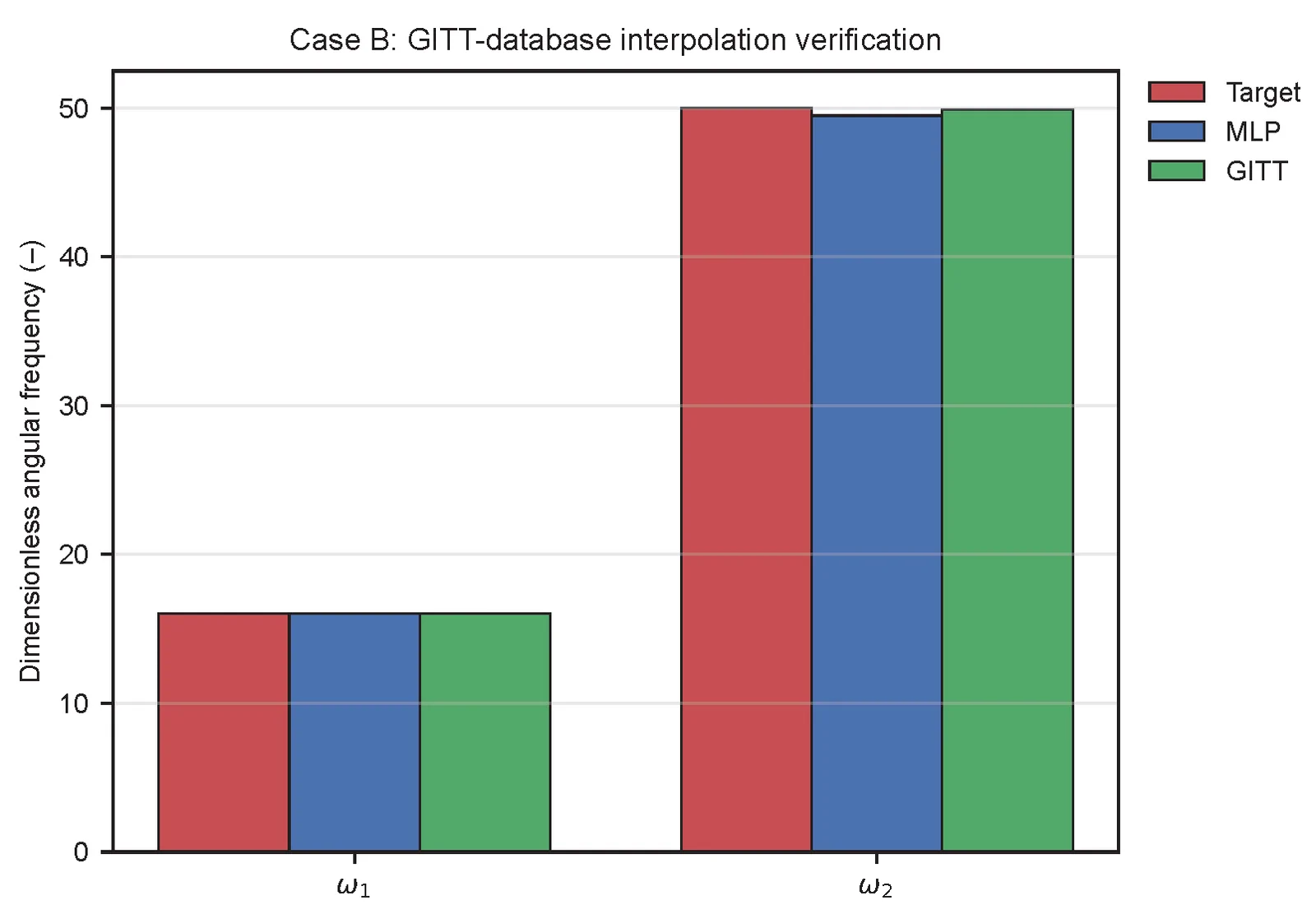
Case B: GITT-database interpolation verification of the target and MLP predictions for $\omega_{1}$ and $\omega_{2}$ (PSO design).

GITT-database interpolation verification at $k^{★}=0.39$ (PSO design) yields $\omega_{1}^{\mathrm{GITT},\mathrm{db}-\mathrm{int}}=16.03$ and $\omega_{2}^{\mathrm{GITT},\mathrm{db}-\mathrm{int}}=49.88$, i.e., target-relative residuals of 0.19% and 0.25%, respectively. This agreement indicates local consistency between the surrogate candidate and the interpolated database reference in the examined pre-critical regime; it is not an independent GITT recalculation. Figure 6 provides a visual comparison of the target, MLP, and database-interpolated GITT values for both modal frequencies.

### 4.3. Case C: Deflection-Aware Design

**Figure 7:**
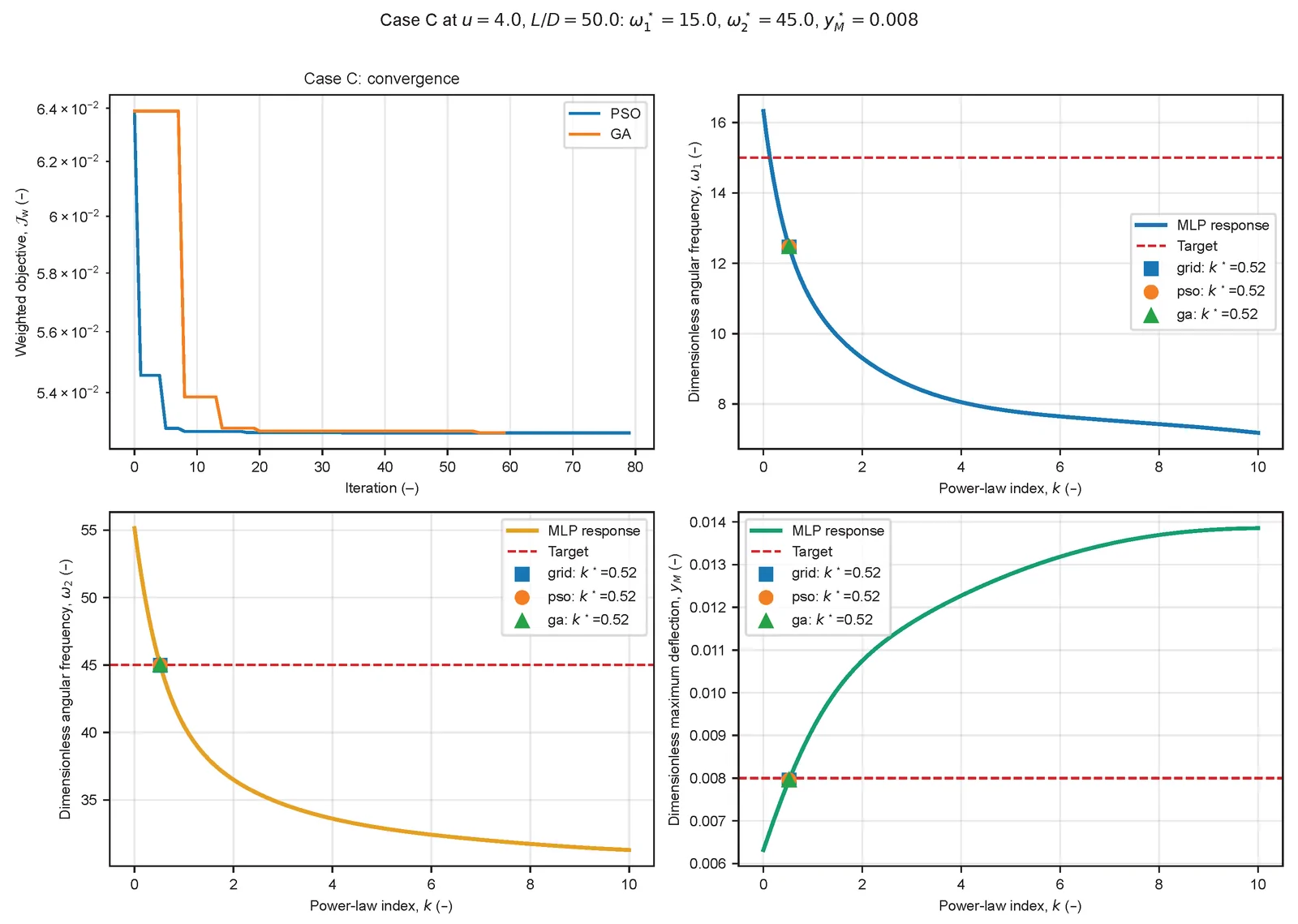
Case C: optimizer convergence and MLP forward maps for $\omega_{1}$, $\omega_{2}$, and $y_{M}$ with triplet targets at u=4.0, L/D=50.

Case C specifies the triplet $\left(\omega_{1}^{★},\omega_{2}^{★},y_{M}^{★}\right)=\left(15.0,45.0,0.0080\right)$ at $u_{0}=4.0$, ${\left(L/D\right)}_{0}=50$, with weights (0.3,0.3,0.4) emphasizing deflection. Figure 7 shows optimizer convergence and MLP forward maps.

**Figure 8:**
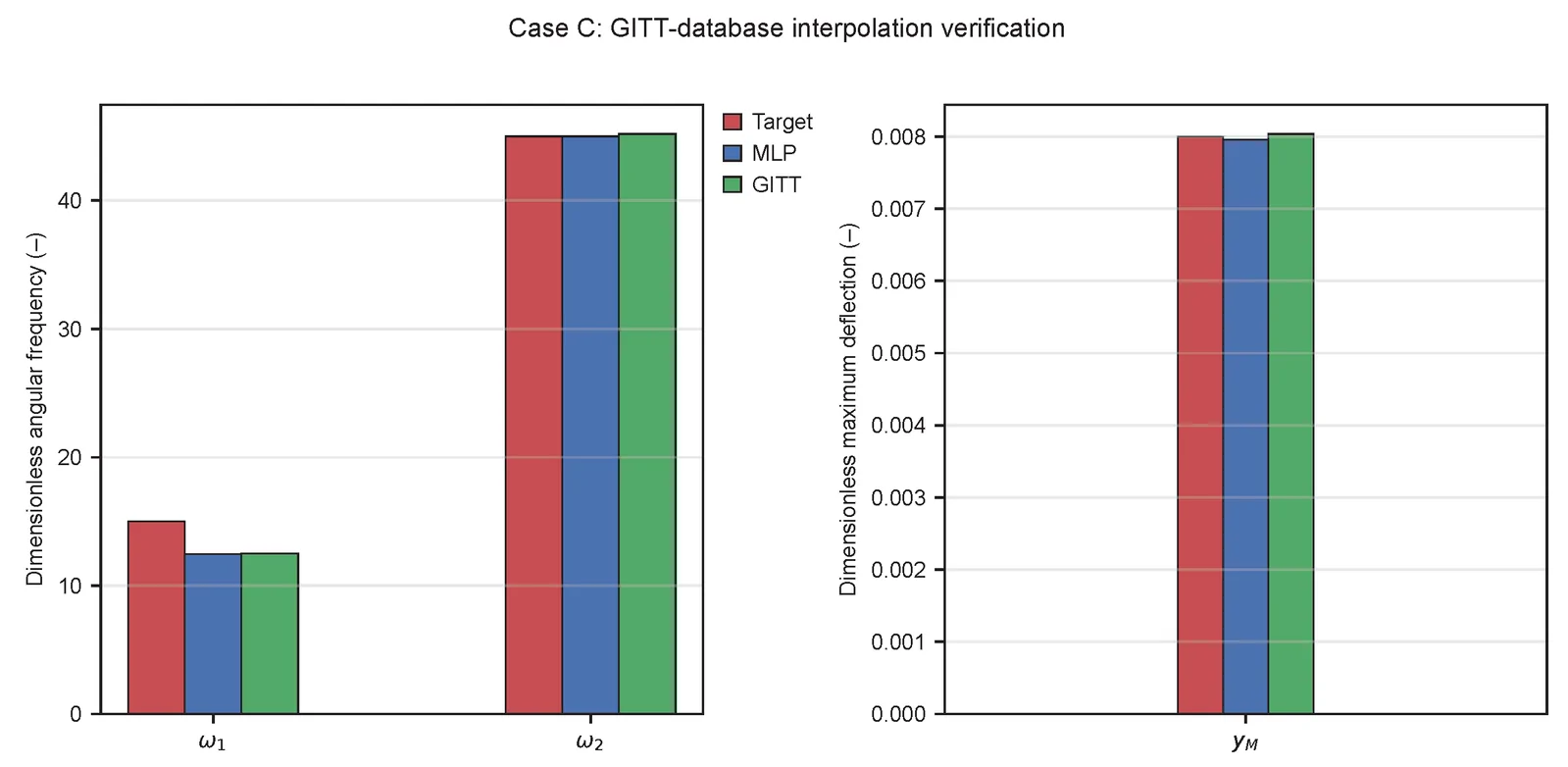
Case C: GITT-database interpolation verification of target and MLP values (PSO design).

All three solvers converge to $k^{★}\approx 0.52$ with ${\hat{y}}_{M}\approx 0.0080$ closely matched. However, ${\hat{\omega }}_{1}\approx 12.5$ falls short of $\omega_{1}^{★}=15.0$ because the simultaneous triplet lies near the edge of the achievable response envelope at $u_{0}=4.0$. GITT-database interpolation verification at $k^{★}=0.5192$ gives ${\left(\omega_{1},\omega_{2},y_{M}\right)}^{\mathrm{GITT},\mathrm{db}-\mathrm{int}}=\left(12.513,45.191,0.008038\right)$, with target-relative residuals of 16.58%, 0.43%, and 0.47%, respectively. This outcome illustrates the practical need for feasibility checks and motivates the Pareto formulation in Case D. Figure 8 compares the target, MLP, and database-interpolated values for the PSO design, confirming that $\omega_{2}$ and $y_{M}$ are closely matched while $\omega_{1}$ remains infeasible.

### 4.4. Case D: Pareto Front

**Figure 9:**
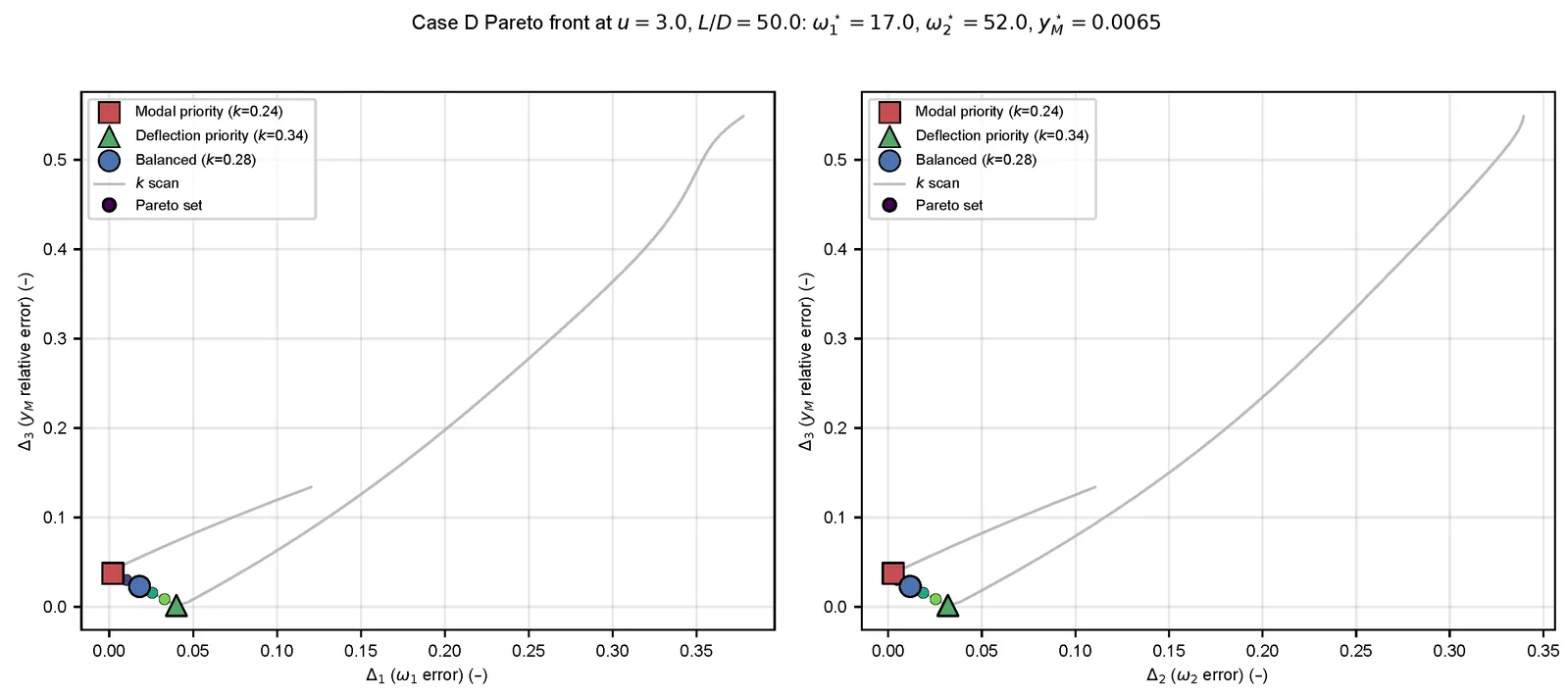
Case D: Pareto trade-offs in the $\left(\Delta_{1},\Delta_{3}\right)$ and $\left(\Delta_{2},\Delta_{3}\right)$ planes. The thin solid gray curve connects all 501 designs in ascending *k* order and, therefore, shows the complete grid-scan trajectory; the small colored points are the non-dominated subset. The square, triangle, and circle identify the modal-priority, deflection-priority, and balanced representative designs, respectively.

Case D specifies $\left(\omega_{1}^{★},\omega_{2}^{★},y_{M}^{★}\right)=\left(17.0,52.0,0.0065\right)$ at $(u_{0},{\left(L/D\right)}_{0})=\left(3.0,50\right)$. Because no single *k* can minimize all three deviations simultaneously, 501-point grid evaluation followed by non-dominated sorting yields the Pareto set in Figure 9. Note that the axes in Figure 9 show the MLP-based deviations $\Delta_{i}$ (Equation (6)), whereas the numerical values quoted below are the GITT-database-interpolated residuals $\varepsilon_{i}^{\mathrm{db}-\mathrm{int}}$ (Equation (8)); the two are closely correlated, with the database-interpolated residuals typically slightly larger than $\Delta_{i}$ due to residual MLP approximation error. Three representative solutions illustrate the conflict:

- **Modal priority** ($k^{★}=0.24$): $\varepsilon_{1}=1.0\%$, $\varepsilon_{2}=1.5\%$, but $\varepsilon_{3}=5.7\%$.
- **Deflection priority** ($k^{★}=0.36$): $\varepsilon_{3}=0.5\%$, but $\varepsilon_{1}=4.7\%$, $\varepsilon_{2}=3.2\%$.
- **Balanced** ($k^{★}=0.28$): all three errors below 3% ($\varepsilon_{1}=1.8\%$, $\varepsilon_{2}=0.9\%$, $\varepsilon_{3}=2.6\%$).

**Figure 10:**
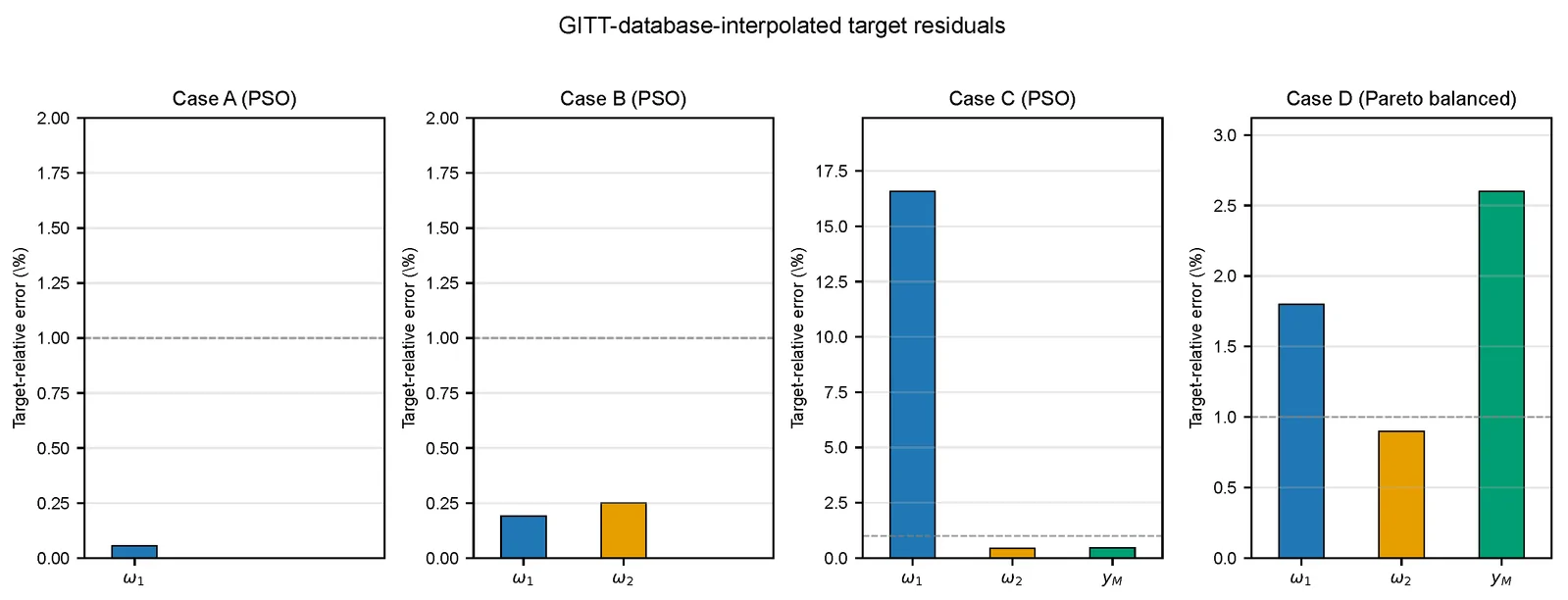
GITT-database-interpolated target-relative errors for Cases A–D (PSO designs for A–C; balanced Pareto design for D). The horizontal dashed line denotes a 1% target-relative-error reference threshold. Response colors are standardized throughout the manuscript: blue for $\omega_{1}$, orange for $\omega_{2}$, and green for $y_{M}$.

All three designs are subjected to GITT-database interpolation verification at the respective $k^{★}$ values. Figure 10 summarizes the database-interpolated target residuals across Cases A–D.

### 4.5. Case E: Two-Variable Optimization of (k,u)

To illustrate the scalability of the framework beyond single-variable inversion, Case E targets $\omega_{1}^{★}=17.0$ at fixed ${\left(L/D\right)}_{0}=50$ by simultaneously optimizing both *k* and *u* via Equation (4). Weights are $\left(w_{1},w_{2},w_{3}\right)=\left(1,0,0\right)$.

**Figure 11:**
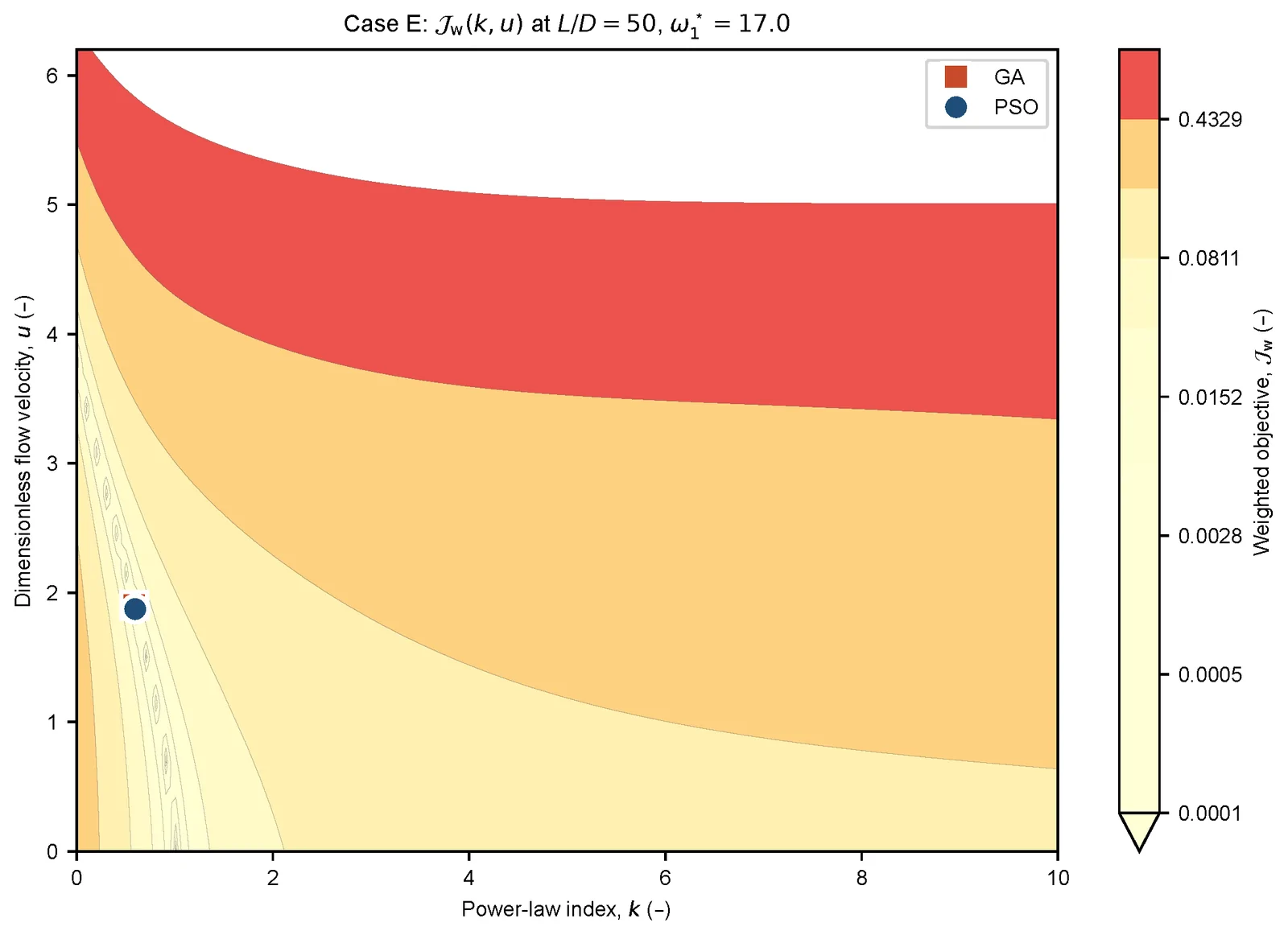
Case E: two-variable (k,u) optimization. Contours of $J_{w}\left(k,u\right)$ from MLP evaluated on a 100×100 grid; PSO and GA optima are marked.

Figure 11 shows the objective function $J_{w}\left(k,u\right)$ computed from MLP evaluations on a 100×100 grid, with the PSO and GA optima marked. Both optimizers converge to $k^{★}\approx 0.59$–0.60 and $u^{★}\approx 1.87$–1.90, representing a distinctly different design from Case A ($k^{★}=0.23$, $u_{0}=3.0$ fixed). While both designs achieve $\omega_{1}=17.0$ on the MLP surrogate, the Case E solution accomplishes this at reduced flow velocity with a steeper gradation ($k^{★}$ increased from 0.23 to 0.60), illustrating the trade-off between material tuning and flow conditions. Table 7 compares this two-variable result with the Case A single-variable optimum.

The frequency-only inverse mapping is non-unique: within the sampled domain, a continuous locus of near-zero-residual (k,u) pairs can reproduce ${\hat{\omega }}_{1}\approx 17.0$ at L/D=50. Accordingly, $\left(k^{★},u^{★}\right)\approx \left(0.60,1.87\right)$ is reported as one feasible surrogate solution returned by PSO under the stated seed and search settings, not as a unique or physically preferred optimum. A unique engineering selection would require an explicitly specified secondary criterion such as minimum flow velocity, minimum deflection, stability margin, manufacturability, or distance from a reference design.

### 4.6. Case F: Two-Variable Optimization of (k,L/D)

**Figure 12:**
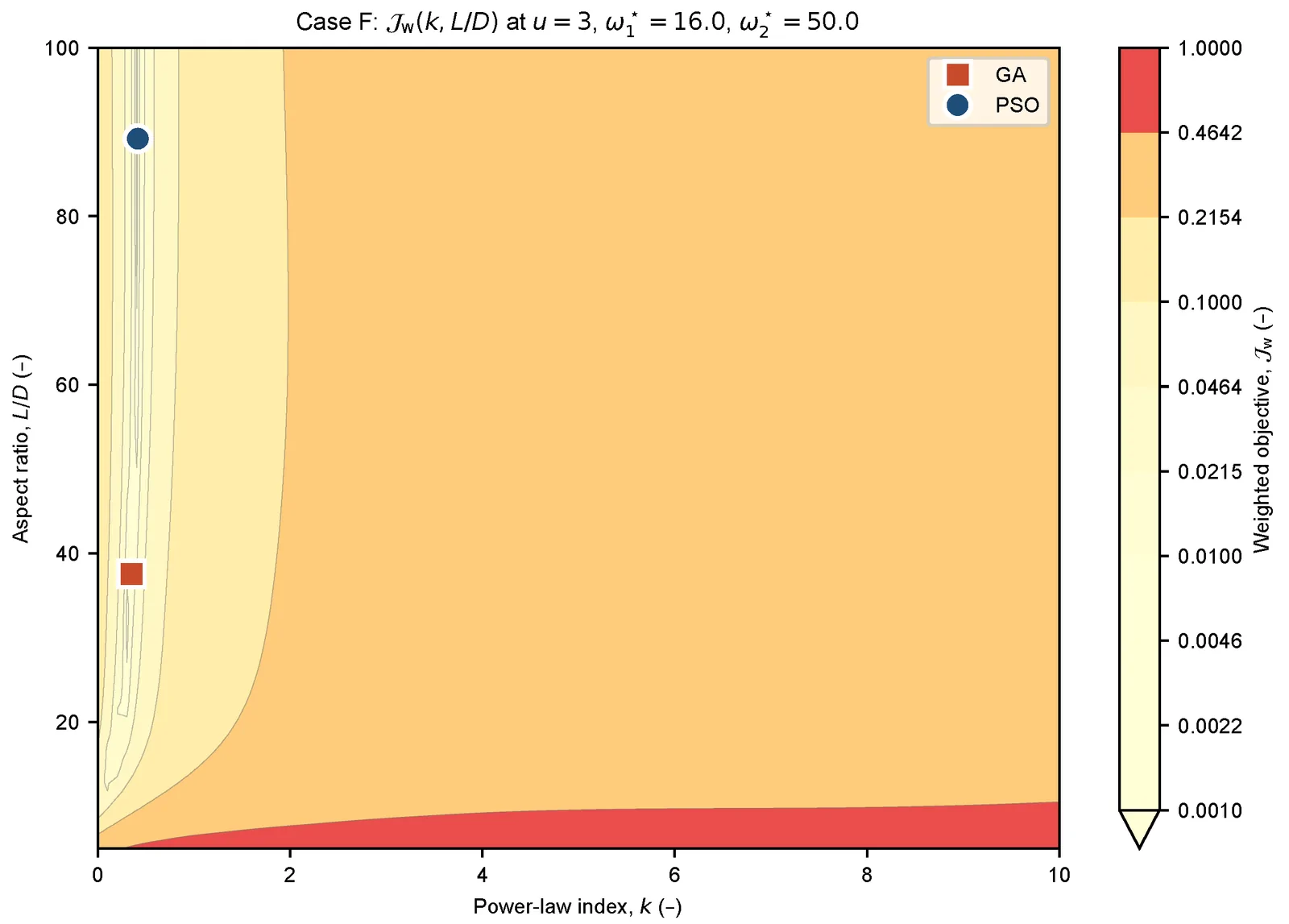
Case F: two-variable (k,L/D) optimization. Contours show the composite objective $J_{w}\left(k,L/D\right)$ with dual-modal targets. PSO converges near (0.42,89.2), whereas GA converges to the distinct higher-objective solution near (0.35,37.6).

Case F simultaneously optimizes (k,L/D) for dual-modal targets $\left(\omega_{1}^{★},\omega_{2}^{★}\right)=\left(16.0,50.0\right)$ at fixed $u_{0}=3.0$, using Equation (5) with weights (0.5,0.5,0). Figure 12 maps the composite objective $J_{w}\left(k,L/D\right)$ and marks the PSO and GA optima.

The PSO optimizer converges to $(k^{★},{\left(L/D\right)}^{★})\approx \left(0.42,89.2\right)$, achieving ${\hat{\omega }}_{1}=16.00$ and ${\hat{\omega }}_{2}=50.00$ ($J_{w}\approx 0$). Because ${\left(L/D\right)}^{★}=89.2$ does not coincide with a database node, no full GITT-database interpolation verification is claimed for this continuous point. The normalized-distance rule selects the database node (u,k,L/D)=(3.0,0.5,99), whose response (15.359,48.171,0.006838) provides modal target residuals of 4.01% and 3.66%; these values are proximity assessments rather than responses at the continuous optimum. GA instead returns a distinct higher-objective solution $(k^{★},{\left(L/D\right)}^{★})\approx \left(0.35,37.6\right)$ with $J_{w}\approx 0.005$. The contour map shows an elongated, weakly varying low-objective valley rather than a single sharply isolated minimum. Because PSO and GA use different stochastic updates, finite populations, random initialization, and stopping criteria, they can terminate at different locations in this valley. Thus, neither coordinate pair is interpreted as a uniquely identified design. Compared with Case B ($k^{★}=0.39$, L/D=50 fixed, $\varepsilon_{1}^{\mathrm{db}-\mathrm{int}}=0.21\%$, $\varepsilon_{2}^{\mathrm{db}-\mathrm{int}}=0.23\%$), the two-variable solutions demonstrate the additional design flexibility obtained by freeing L/D, but their database-level physical assessment remains approximate.

### 4.7. Case G: Inactive-Constraint Consistency Check

Case G is retained only as a consistency check for the constrained formulation of Equation (7). It targets $\omega_{1}^{★}=17.0$ at $(u_{0},{\left(L/D\right)}_{0})=\left(3.0,50\right)$ with ${\hat{y}}_{M}\leq 0.008$. The PSO algorithm minimizes $|\omega_{1}^{★}-{\hat{\omega }}_{1}\left(k\right)|$ with a penalty that activates when ${\hat{y}}_{M}\left(k\right)>0.008$.

**Figure 13:**
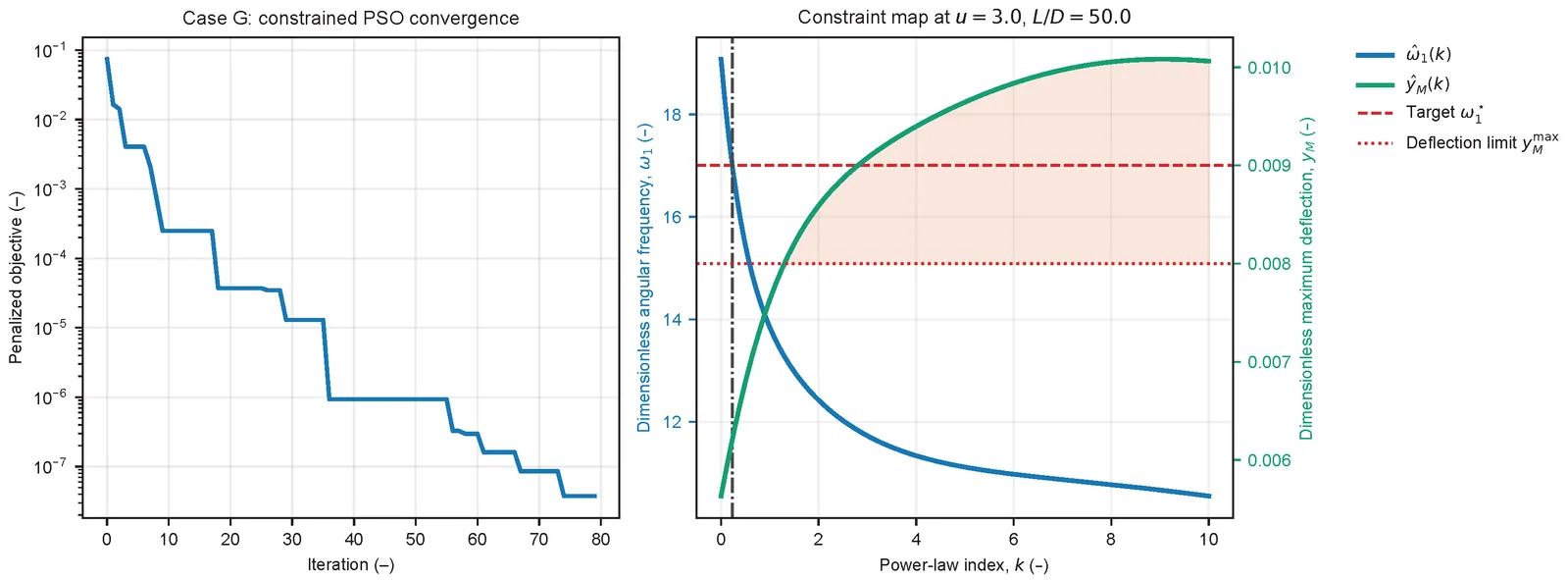
Case G: inactive-constraint consistency check. (**Left**): PSO convergence. (**Right**): the blue and green curves denote ${\hat{\omega }}_{1}\left(k\right)$ and ${\hat{y}}_{M}\left(k\right)$, respectively; the red dashed and dotted lines denote the frequency target and deflection limit; the red shading denotes the infeasible region $y_{M}>0.008$; and the black dash-dotted line marks the optimized $k^{★}$. The Case A optimum already satisfies the bound, so the constraint remains inactive and the solution is unchanged.

Figure 13 shows the PSO convergence history and the MLP forward curves ${\hat{\omega }}_{1}\left(k\right)$ and ${\hat{y}}_{M}\left(k\right)$ with the infeasible region ($y_{M}>0.008$) shaded. At u=3.0, the unconstrained optimum $k^{★}\approx 0.23$ (Case A) already satisfies the deflection bound (${\hat{y}}_{M}\approx 0.0062<0.008$), so the constraint is inactive; the PSO converges to the same $k^{★}=0.23$ as Case A. GITT-database interpolation at $k^{★}=0.2303$ gives $\omega_{1}^{\mathrm{GITT},\mathrm{db}-\mathrm{int}}=16.991$ and $y_{M}^{\mathrm{GITT},\mathrm{db}-\mathrm{int}}=0.006206$.

Because the bound is inactive, this example confirms only that the constrained implementation reduces to the unconstrained solution when the penalty is not activated; it is not presented as evidence of an active constrained optimum.

## 5. GITT-Database Interpolation Verification and Computational Performance

### 5.1. GITT-Database Interpolation Verification Results

**Figure 14:**
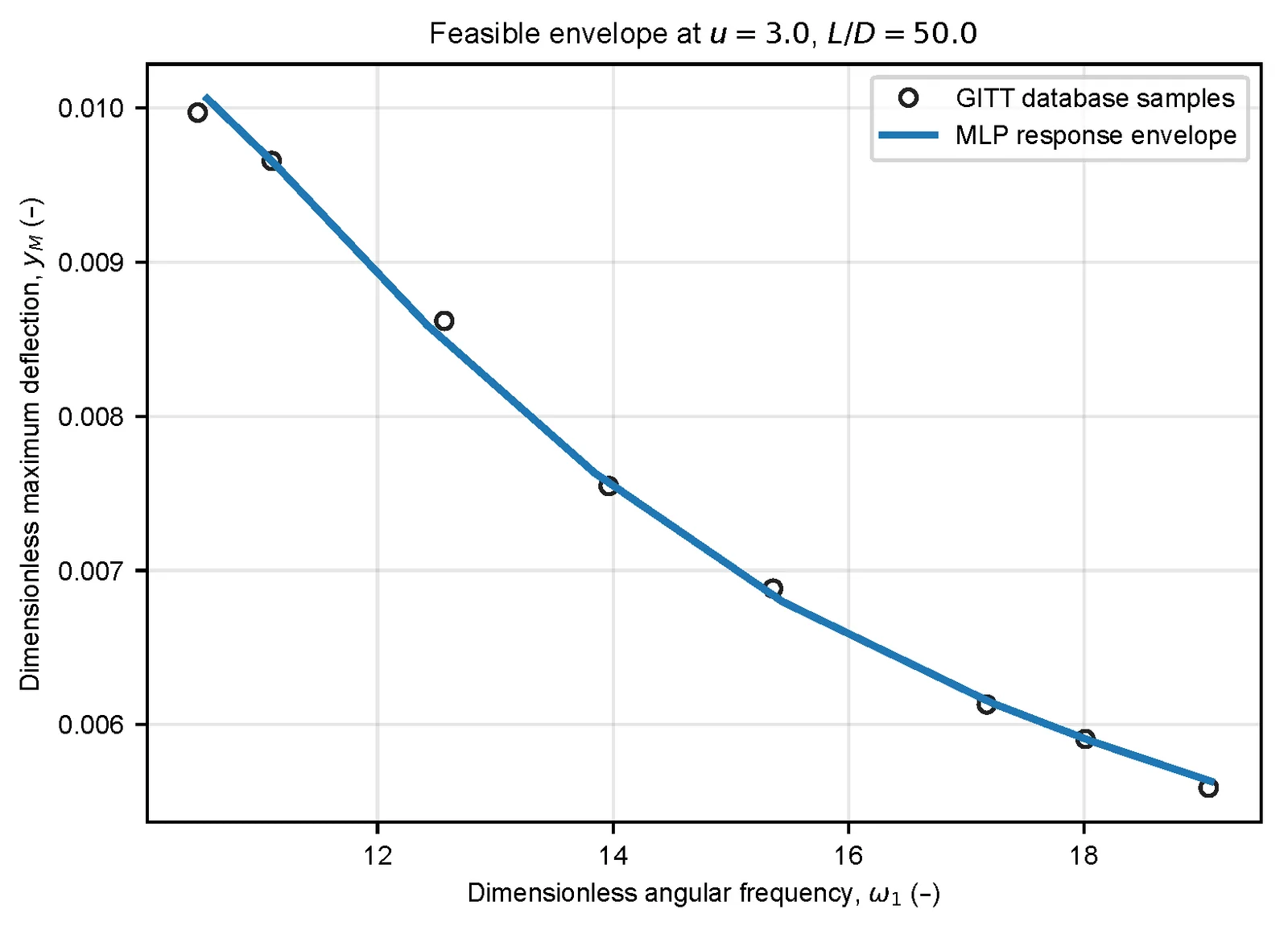
Achievable $\left(\omega_{1},y_{M}\right)$ response envelope at fixed operating conditions (Case A).

Table 8 summarizes the database-based assessment. Cases A–D and G lie on sampled (u,L/D) slices and can therefore be evaluated by one-dimensional interpolation along *k*. Cases E and F contain an off-grid optimized variable; their nearest-node responses are labeled “Provisional,” a database-assessment status rather than independent engineering-feasibility confirmation. Among the single-variable target-matching cases, Cases A and B give database-interpolated target residuals of 0.055% and 0.21–0.23%, respectively. Case C is partially infeasible: $\omega_{2}$ and $y_{M}$ are recovered within 1%, whereas $\omega_{1}$ cannot be met at $u_{0}=4.0$ for k∈[0,10]. Figure 14 illustrates the achievable $\left(\omega_{1},y_{M}\right)$ envelope at the Case A operating point. No entry in Table 8 is labeled as direct GITT verification because no new GITT calculation was performed.

### 5.2. Computational Performance

**Figure 15:**
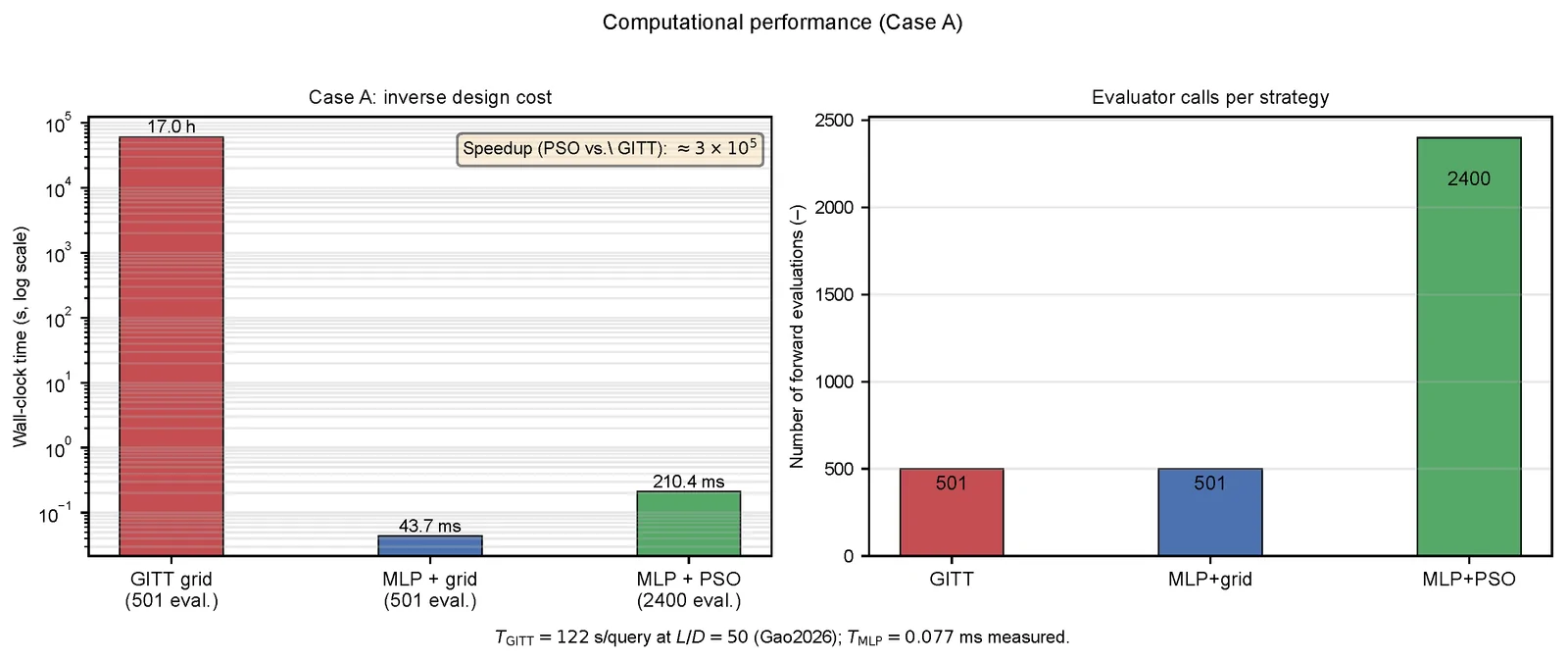
Computational performance comparison: GITT brute-force versus MLP-assisted strategies (log scale); right panel shows forward-evaluation counts.

Table 9 and Figure 15 compare the wall-clock cost of Case A inverse design. A brute-force GITT grid search over 501 *k* values would require approximately $6.1\times 10^{4}$ s (≈17 h), extrapolated from the measured single-query time $T_{\mathrm{GITT}}\approx 122$ s at L/D=50 [14]. In the synchronized Table 9/Figure 15 benchmark, raw MLP inference, MLP + 1D grid, and MLP + PSO required 0.077 ms/query, $4.37\times 10^{-2}$ s, and $2.104\times 10^{-1}$ s. End-to-end optimizer times include preprocessing, all MLP calls, objective evaluation, and bookkeeping, but exclude database comparison. Table 1’s 0.085 ms/query is retained only as the published Ref. [14] benchmark and is not used in the synchronized timings.

The reported online comparison excludes the one-time offline construction cost. The archived logs do not contain a measured aggregate wall time for all 336 heterogeneous GITT cases. Using the published representative mean of 47 s per case yields a clearly labeled estimate of $336\times 47\;s\approx 1.58\times 10^{4}$ s (≈4.4 h), plus approximately 0.77 s to train the three MLPs [14]. Because individual GITT runtimes vary with L/D (approximately 10–122 s per case), 4.4 h is a hardware- and case-dependent order-of-magnitude estimate. The database can be reused for new inverse targets within the same validated domain; changing the material system, boundary condition, forward model, or parameter domain requires new database generation.

## 6. Discussion

### 6.1. Physical Interpretation

In the adopted Alumina–Steel gradation, the left end is alumina-rich ($E_{L}>E_{R}$, $\rho_{L}<\rho_{R}$) and the right end is steel-rich. Increasing *k* keeps effective properties closer to the left-end values over most of the span, reducing global bending stiffness and lowering $\omega_{1}$ and $\omega_{2}$ [14]. The inverse results mirror this physics: Case A ($\omega_{1}^{★}=17.0$) selects the intermediate $k^{★}=0.23$, whereas Case B’s lower fundamental target ($\omega_{1}^{★}=16.0$) and explicit $\omega_{2}^{★}=50.0$ requirement shift the optimum to $k^{★}=0.39$, reflecting the stronger influence of *k* on $\omega_{2}$. The dimensionless flow velocity *u* remains the dominant driver of modal frequencies [8,28]; the two-variable solutions in Cases E and F confirm that when *u* or L/D are freed as decision variables, the optimizer trades between *k* and the geometric/flow parameter to achieve targets, producing a continuum of feasible designs.

### 6.2. Single-Variable vs. Two-Variable Optimization

The single-variable cases (A–D) demonstrate that optimizing *k* alone is sufficient when $u_{0}$ and ${\left(L/D\right)}_{0}$ are tightly constrained by operational requirements (e.g., a fixed flow rate and available riser length). The two-variable extensions (Cases E–F) address scenarios where some flexibility in flow velocity or aspect ratio exists. The non-uniqueness of the (k,u) mapping (Case E, Figure 11) means that additional criteria—minimum $y_{M}$, maximum buckling margin, or manufacturing constraints—should be imposed to select a unique design from the isocontour. Both extensions maintain the same MLP + PSO architecture with trivial modifications to the search-space bounds, confirming that the framework scales to higher-dimensional design spaces without methodological changes.

### 6.3. Engineering Implications

Conventional gradation design for fluid-conveying pipelines often relies on repeated high-fidelity runs or laboratory iterations over *k* under fixed operating conditions, which is costly when each GITT query requires $O(10^{2})$ s [14]. The proposed closed loop reduces the online search to $O(10^{2})$–$O(10^{3})$ millisecond-scale MLP evaluations and returns a candidate design within sub-second wall-clock time, followed, where the candidate lies on a sampled (u,L/D) slice, by GITT-database interpolation verification. This database comparison is a screening step rather than an independent high-fidelity certification. For oil-and-gas risers, subsea jumpers, and aerospace propellant lines—where modal clearance and vibration amplitude must be met at specified flow rates—this workflow offers a practical route from target performance specifications to gradation indices without exhaustive trial-and-error [4,5,18].

### 6.4. Sensitivity-Driven Interpretation of Inverse Designs

**Figure 16:**
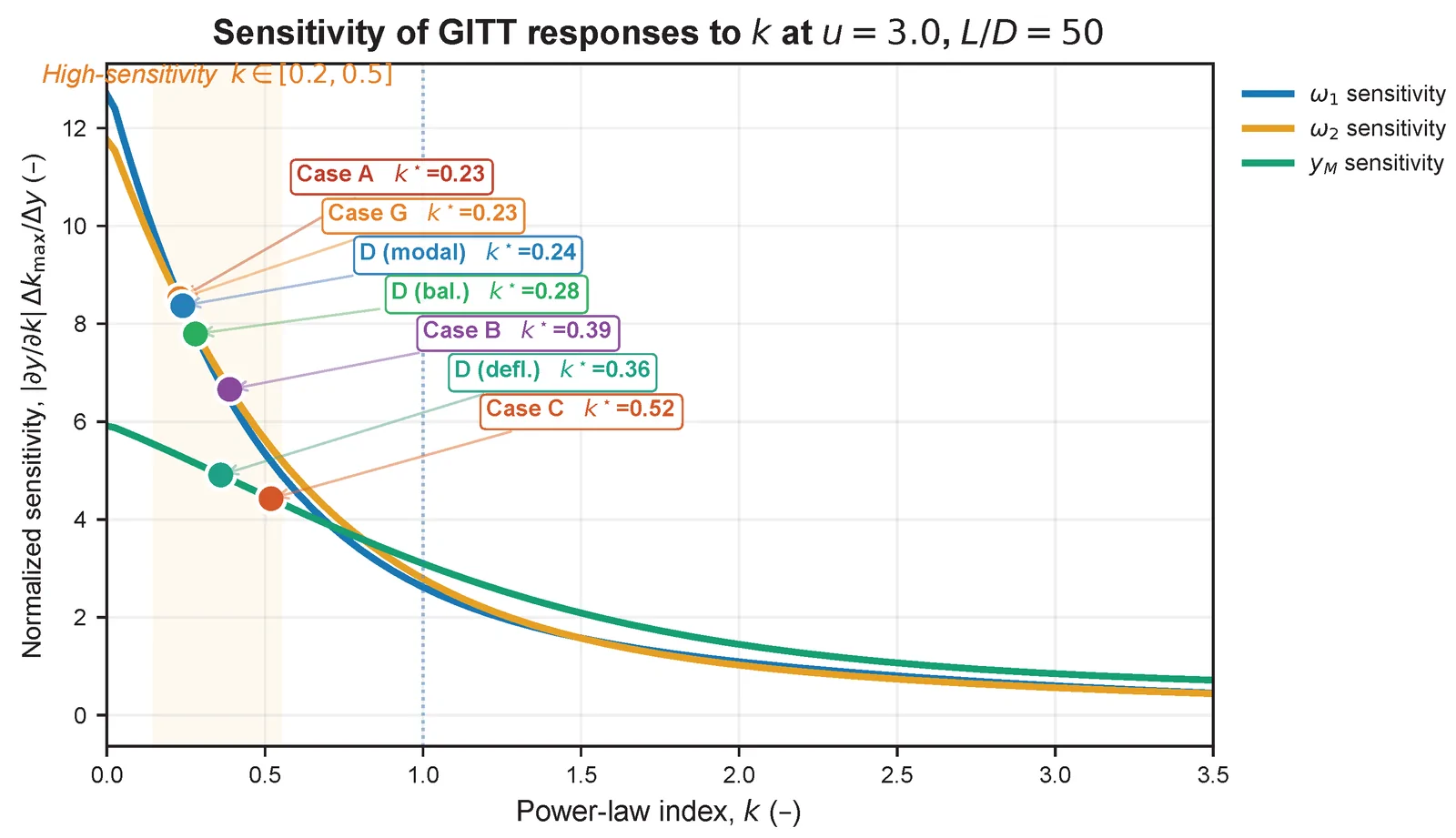
Normalized sensitivity of $\omega_{1}$, $\omega_{2}$, and $y_{M}$ to the power-law index *k*, computed via the trained MLP surrogate at u=3.0, L/D=50. Optimized $k^{★}$ values from Cases A–D and G are marked, all falling within the high-sensitivity region k∈[0.2,0.5].

Figure 16 overlays the MLP-computed normalized sensitivity of $\omega_{1}$, $\omega_{2}$, and $y_{M}$ with respect to *k* at the nominal operating point (u=3.0,L/D=50), together with the optimized $k^{★}$ values from Cases A–D and G. The normalized sensitivity is defined as $|\partial y/\partial k|\cdot (k_{\max}-k_{\min})/\Delta y$, which quantifies the relative importance of *k* for each response over the design domain.

Each marker is placed on the sensitivity curve corresponding to that case’s primary optimization objective ($\omega_{1}$ for Cases A, G, and D-modal/balanced; $\omega_{2}$ for Case B; $y_{M}$ for Cases C and D-deflection-priority), so that the dot naturally sits in the region where the optimizer is most sensitive to the target response. The optimized $k^{★}$ values consistently cluster in the interval k∈[0.2,0.5], which coincides with the region of maximum $\omega_{1}$ and $y_{M}$ sensitivity. This confirms that the inverse design framework naturally directs the optimizer toward the *k*-range where material gradation exerts the strongest leverage on the target responses—a physically intuitive outcome that reinforces the credibility of the MLP-guided search. The $\omega_{2}$ sensitivity peaks at slightly higher *k* (k≈1), which explains why the dual-modal Case B selects a larger $k^{★}=0.39$ than the single-$\omega_{1}$ Case A ($k^{★}=0.23$): the optimizer must balance $\omega_{1}$ accuracy (favored at low *k*) with $\omega_{2}$ accuracy (favored where $\omega_{2}$ is more sensitive).

### 6.5. Training-Repeatability Assessment

**Figure 17:**
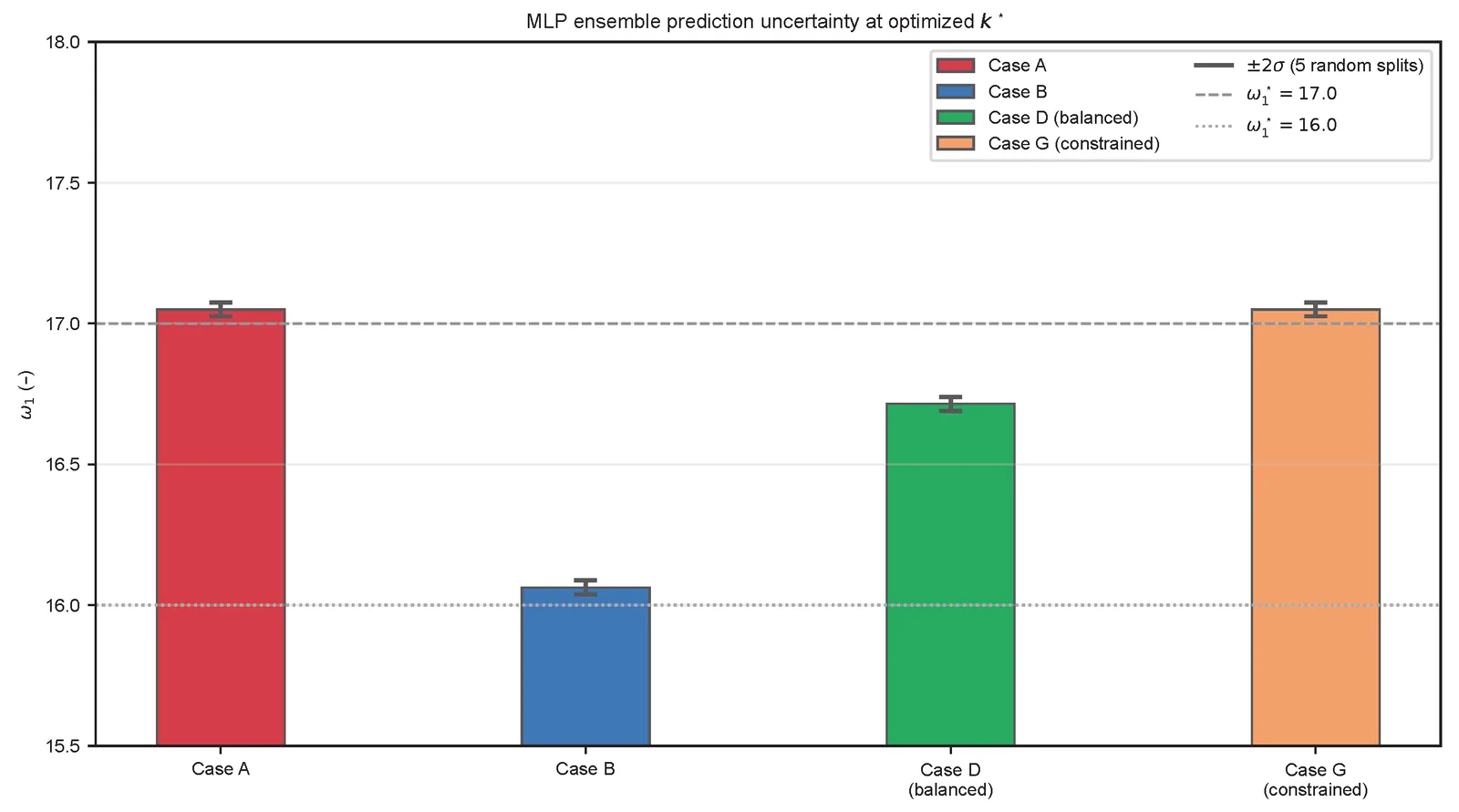
Training-repeatability assessment: MLP predictions (±2σ) from $N_{\mathrm{ens}}=5$ runs with different data partitions at the reported $k^{★}$ values. Target values are marked by horizontal lines. The bars are descriptive inter-predictor spreads, not calibrated confidence intervals or predictive-uncertainty bounds, and exclude database, interpolation, model-form, and extrapolation uncertainty.

Figure 17 summarizes one specific source of surrogate variability using an ensemble of $N_{\mathrm{ens}}=5$ MLP predictors trained with different random train–test partitions. At each reported $k^{★}$ (Cases A, B, D-balanced, and G), the inter-predictor standard deviation of $\omega_{1}$ was approximately 0.012. This small spread indicates repeatability with respect to the sampled data partitions and training randomness considered here. It is not a complete predictive-uncertainty estimate: uncertainty in the GITT database, database interpolation, model-form assumptions, measurement inputs, and extrapolation is not represented. With only five predictors, the plotted ±2σ bars describe inter-predictor spread; they are not calibrated confidence intervals or predictive-uncertainty bounds and do not establish inverse-design reliability.

### 6.6. Limitations

Several limitations should be noted. (i) Predictions outside the training hyper-rectangle k∈[0,10],u∈[0,6.2],L/D∈[5,100] are unreliable. (ii) Multi-target conflicts may prevent exact simultaneous matching; Pareto fronts should be reported rather than overstating single-*k* optimality, as demonstrated in Case D. (iii) The present verification is based on interpolation within the same 336-sample GITT database used to develop the surrogate. It is useful for checking local database consistency but is not independent, does not provide a high-fidelity error bound, and does not replace a new GITT calculation at a candidate design. For Cases E and F, the second optimized variable is off-grid and only nearest-node assessment is reported. (iv) The two-variable cases are demonstrated for dual-modal targets; extension to three-output (k,u,L/D) optimization would require additional constraints to maintain problem well-posedness. (v) The nominal-geometry model does not resolve manufacturing tolerances, ovality, wall-thickness variation, internal roughness, pressure loss, temperature-dependent properties, or a change of flow regime. These effects should be incorporated through a coupled hydraulic/thermal model and updated structural parameters before applying the inverse design to an as-built pipe. (vi) No explicit independent damping term is included. Thus, the calculated values are undamped natural frequencies; adding material, viscous, support, or coating damping would generally produce complex eigenvalues and should be considered when damped resonance amplitudes or decay rates are design targets. The ensemble analysis quantifies only sensitivity to MLP training randomness and does not cover these model-form uncertainties.

## 7. Conclusions

A surrogate-assisted inverse design framework for the power-law gradation index of clamped–clamped AFG Timoshenko pipes conveying fluid has been established. The previously published GITT database and MLP forward surrogate [14] are reused, while the present contribution is the inverse formulation, optimizer comparison, feasibility analysis, and database-based assessment. Candidates on sampled (u,L/D) slices are checked by GITT-database interpolation verification; the two off-grid two-variable candidates receive only nearest-node assessment. No new GITT calculation is claimed. The main findings are as follows:

1. For single-variable *k* optimization, Cases A and B give GITT-database-interpolated target-relative residuals of 0.055% and 0.21–0.23%, respectively, with cross-optimizer agreement among grid, PSO, and GA.
2. Case C demonstrates accurate inversion of $\omega_{2}$ and $y_{M}$ (<1%) but reveals a feasibility limit for $\omega_{1}$ at $u_{0}=4.0$, highlighting the need for feasibility checks.
3. Case D confirms the expected modal–deflection Pareto trade-off, with a balanced design achieving sub-3% errors on all three targets.
4. Two-variable extensions (Case E: (k,u); Case F: (k,L/D)) demonstrate surrogate-level exploration of higher-dimensional design spaces. Their continuous optima are provisional because only nearest-node database assessments are available.
5. Case G is an inactive-constraint consistency check: its bound is satisfied, but it does not demonstrate an active constrained optimum. The five-model ensemble gives an inter-predictor standard deviation of approximately 0.012 for $\omega_{1}$ and assesses training/partition repeatability only. The sensitivity bridge analysis shows that all optimized $k^{★}$ values fall naturally within the high-sensitivity region k∈[0.2,0.5], providing a physics-based rationale for the inverse design results.
6. For Case A, the synchronized MLP grid and PSO benchmarks require 0.044 and 0.210 s, respectively, compared with an extrapolated 17 h GITT grid.

Future work will extend the ensemble UQ framework into robust optimization under uncertainty [29], extend the framework to three-variable (k,u,L/D) optimization under additional geometric and stability constraints, and explore gradient-enhanced or physics-informed surrogates for higher-dimensional material distribution parametrizations [23,30].

## Figures and Tables

**Table 1:** MLP surrogate accuracy (reproduced from [14]).

Output	$R^{2}$ (Test)	$\varepsilon_{\max}$ (%)	$N_{\mathrm{tr}}$	$T_{\mathrm{MLP}}$ (ms)
$\omega_{1}$	0.9982	8.90	270	0.085
$\omega_{2}$	0.9995	2.25	270	0.085
$y_{M}$	0.9911	8.89	270	0.085

**Table 2:** Principal symbols and units used in the forward and inverse formulations.

Symbol	Definition	Unit
*k*	Axial material power-law index	–
*Z*, *L*, *D*, *d*	Axial coordinate, pipe length, outer diameter, inner diameter	m
*U*; *u*	Dimensional; dimensionless prescribed mean flow velocity	m/s; –
*T*; *t*	Dimensional; dimensionless time	s; –
*Y*; *y*, $y_{M}$	Dimensional; dimensionless displacement and its maximum	m; –
$\rho_{f}$, $m_{f}$	Fluid density and fluid mass per unit length	kg/m^3^; kg/m
$E_{0}$, $\rho_{0}$, $A_{p}$, $I_{p}$	Reference modulus, density, wall area, second moment of area	Pa; kg/m^3^; m^2^; m^4^
$\omega_{i}$; $\Omega_{i}$; $f_{i}$	Dimensionless angular; dimensional angular; cyclic frequency	–; rad/s; Hz
x, y, $\hat{y}$	Design vector, response vector, and MLP-predicted response	–
$w_{i}$, $\Delta_{i}$, $J_{w}$	Objective weight, relative deviation, weighted objective	–
$\tau_{i}$, $\varepsilon_{i}$	Constraint tolerance and target-relative error	–; %

**Table 3:** Comparison with the published homogeneous, zero-flow, clamped–clamped GITT benchmark of An and Su [27]. Both studies use the same nondimensional frequency definition.

Response	Present Timoshenko–GITT	An and Su [27]	Relative Difference (%)
$\omega_{1}$	22.2006	22.373	0.77
$\omega_{2}$	60.950	61.673	1.17

**Table 4:** Optimizer settings for MLP-assisted inverse design.

Parameter	Grid (1D)	PSO	GA
Search range	k∈[0,10]; 2D: u∈[0,6.2] or L/D∈[5,100]		
Population/grid size	501	30	40
Iterations/generations	1 pass	80	60
PSO: χ, $c_{1}$, $c_{2}$	–	0.729, 1.494, 1.494	–
GA: crossover/mutation	–	–	blend/$p_{\mathrm{mut}}=0.15$
Elitism	–	–	top 2 retained
Random seed	–	42	42

**Table 5:** Case setup for single-variable (*k* only) inverse design.

Case	Description	$u_{0}$	${\left(L/D\right)}_{0}$	Targets $\left(\omega_{1}^{★},\omega_{2}^{★},y_{M}^{★}\right)$	Weights $\left(w_{1},w_{2},w_{3}\right)$
A	Single-target $\omega_{1}$ inversion	3.0	50	(17.0,−−,−−)	(1,0,0)
B	Dual-target $\omega_{1}$–$\omega_{2}$ match	3.0	50	(16.0,50.0,−−)	(0.5,0.5,0)
C	Deflection-aware triplet target	4.0	50	(15.0,45.0,0.0080)	(0.3,0.3,0.4)
D	Pareto trade-off (modal vs. $y_{M}$)	3.0	50	(17.0,52.0,0.0065)	Pareto
G	Constrained $\omega_{1}$ ($y_{M}\;\leq \;0.008$)	3.0	50	(17.0,−−,≤0.008)	(1,0,0)

**Table 6:** MLP-optimized $k^{★}$ and surrogate target residuals before GITT-database interpolation verification (single-variable Cases A–C).

Case	Optimizer	$k^{★}$	${\hat{\omega }}_{1}$	${\hat{\omega }}_{2}$	${\hat{y}}_{M}$	$J_{w}$
A	Grid	0.24	16.93	52.09	0.00623	0.00410
A	PSO	0.23	17.00	52.28	0.00621	≈0
A	GA	0.23	17.00	52.27	0.00621	$9.8\times 10^{-5}$
B	Grid	0.38	16.05	49.62	0.00655	0.00520
B	PSO	0.39	16.00	49.49	0.00657	0.00507
B	GA	0.39	16.01	49.54	0.00656	0.00511
C	PSO	0.52	12.47	45.00	0.00796	0.05272
D	Balanced	0.28	16.66	51.33	0.00632	–
G	PSO (constr.)	0.23	17.00	52.28	0.00621	≈0

**Table 7:** Comparison of single-variable (Case A) and two-variable (Case E) inverse designs for $\omega_{1}^{★}=17.0$.

Strategy	$k^{★}$	$u^{★}$	Database $\omega_{1}$	$\varepsilon_{1}$ (%)	$J_{w}$
Optimize *k* only (Case A PSO)	0.23	3.0 (fixed)	16.991	0.055	≈0
Optimize (k,u) (Case E PSO)	0.60	1.87	17.453 ^†^	2.67 ^†^	≈0

^†^ Nearest database node (u,k,L/D)=(1.8,0.5,50) in normalized (u,k) design space; not a full interpolation verification at the continuous candidate.

**Table 8:** GITT-database interpolation verification and database-based assessment of the optimized candidates. Here, “1D interpolation” denotes interpolation along *k* at a sampled (u,L/D) slice; Cases E and F are only approximated at nearby database nodes.

Case	$x^{★}$	Database Response $\left(\omega_{1},\omega_{2},y_{M}\right)$	Target Residual (%)	Assessment Method	Status
A	(0.2303,3.0,50)	(16.991,52.313,0.006206)	(0.055,−−,−−)	1D interpolation	Database-consistent
B	(0.39,3.0,50)	(16.03,49.88,0.00660)	(0.19,0.25,−−)	1D interpolation	Database-consistent
C	(0.5192,4.0,50)	(12.513,45.191,0.008038)	(16.58,0.43,0.47)	1D interpolation	Partially infeasible
D	(0.28,3.0,50)	(16.69,51.55,0.00633)	(1.8,0.9,2.6)	1D interpolation	Trade-off candidate
E	(0.60,1.87,50)	$\left({17.453\;}^{†},\;{50.270\;}^{†},\;{0.006260\;}^{†}\right)$	$\left({2.67\;}^{†},\;--,\;--\right)$	Nearest-node approximation	Provisional
F	(0.42,3.0,89.2)	$\left({15.359\;}^{‡},\;{48.171\;}^{‡},\;{0.006838\;}^{‡}\right)$	$\left({4.01\;}^{‡},\;{3.66\;}^{‡},\;--\right)$	Nearest-node approximation	Provisional
G	(0.2303,3.0,50)	(16.991,52.313,0.006206)	(0.055,−−,−−)	1D interpolation	Constraint inactive

^†^ Nearest node (u,k,L/D)=(1.8,0.5,50); ^‡^ nearest node (3.0,0.5,99). Nodes are selected by Euclidean distance after normalizing the two active coordinates by their sampled ranges. These are not direct GITT evaluations or full two-dimensional interpolation verification.

**Table 9:** Synchronized computational-cost comparison for inverse design of *k* (Case A). The GITT grid is extrapolated from $T_{\mathrm{GITT}}=122$ s; both MLP rows are measured end-to-end with the same run of inverse_design.py.

Strategy	Forward Evaluations	Wall-Clock Time
GITT grid search ($N_{k}=501$)	501	$6.11\times 10^{4}$ s (≈17 h) *
MLP + 1D grid (baseline)	501	$4.37\times 10^{-2}$ s
MLP + PSO (30×80)	2400	$2.104\times 10^{-1}$ s

* Extrapolated: $501\times T_{\mathrm{GITT}}$ at L/D=50; not re-run online.

## Data Availability

The original contributions presented in this study are included in the article. Further inquiries can be directed to the corresponding author.
